# Stimulus-induced Epileptic Spike-Wave Discharges in Thalamocortical Model with Disinhibition

**DOI:** 10.1038/srep37703

**Published:** 2016-11-23

**Authors:** Denggui Fan, Suyu Liu, Qingyun Wang

**Affiliations:** 1 School of Mathematics and Physics, University of Science and Technology Beijing, Beijing 100083, P. R. China; 2 Department of Dynamics and Control, Beihang University, Beijing 100191, P. R. China

## Abstract

Epileptic absence seizure characterized by the typical 2–4 Hz spike-wave discharges (SWD) are known to arise due to the physiologically abnormal interactions within the thalamocortical network. By introducing a second inhibitory neuronal population in the cortical system, here we propose a modified thalamocortical field model to mathematically describe the occurrences and transitions of SWD under the mutual functions between cortex and thalamus, as well as the disinhibitory modulations of SWD mediated by the two different inhibitory interneuronal populations. We first show that stimulation can induce the recurrent seizures of SWD in the modified model. Also, we demonstrate the existence of various types of firing states including the SWD. Moreover, we can identify the bistable parametric regions where the SWD can be both induced and terminated by stimulation perturbations applied in the background resting state. Interestingly, in the absence of stimulation disinhibitory functions between the two different interneuronal populations can also both initiate and abate the SWD, which suggests that the mechanism of disinhibition is comparable to the effect of stimulation in initiating and terminating the epileptic SWD. Hopefully, the obtained results can provide theoretical evidences in exploring dynamical mechanism of epileptic seizures.

Epilepsy is a chronic neurological disorder which is generally defined as a condition characterized by spontaneous recurrent seizures. Absence epilepsy, mainly occurring in children^1^,^2^,^3^,^4^,^5^,^6^,^7^, is the type of petit mal-like seizures. Usually, during the absence seizure, patients are transiently deprived of consciousness, whose clinical manifestation is characterized by the periodic 2–4 Hz spike-wave discharges (SWDs). Clinical observations show that SWD discharges are bilaterally synchronous and also well structured^3^,^8^. In particular, several types of seizures in children with absence epilepsy display generalized rhythmic SWD discharges through the electroencephalogram (EEG). Similar to the absence seizures in epilepsy, epileptic tonic and clonic seizures^9^,^10^,^11^,^12^,^13^,^14^ are also the primary generalized seizures which also represent the pathological brain rhythms involving the whole cortical region of humans. Generally, they are the types of grand-mal seizures. In the electroencephalograph (EEG) of patients with epileptic tonic-conic seizures, there first exists a tonic phase characterized by paroxysmal fast activity with high frequency (more than 13 Hz), whose clinical manifestations typically show the tense of muscles^15^, the preictal consciousness and invariably postictal unconsciousness^16^. Furthermore, the EEG also shows that the tonic oscillations can be specifically followed by the evolution into the clonic phase of epileptic seizures with low-frequency and high-amplitude slow-wave oscillation^8^,^17^. Actually, electrophysiological experiments have long revealed the existence of two-way transitions between epileptic absence and tonic-clonic seizures in the cerebral cortex^18^,^19^.

Intuitively, ~2–4 Hz SWD discharges of absence seizure can be benign due to the transient deprivation of consciousness^20^. However, under the recurrent or frequent occurrences of SWD discharges, all kinds of accompanied behavioural, linguistic and cognitive disorders^20^,^21^,^22^ can be observed from the patients with absence seizure. Pharmacotherapy is the conventional treatment for the epileptic disorders^20^,^23^. But, because of the chronic nature of the disorder, patients with anti-epileptic drug treatment often suffer from side-effects, which can chronically impact their quality of life^23^. In addition, because of the absence of neuroradiological abnormalities for the SWD discharges, invasive treatments are frequently and typically not indicated^20^. Alternative therapies to reduce the SWD discharges of absence seizure are need to be further explored. To some extent, stimulus perturbations can control or suppress the epileptic seizures 20,24,25,26,27,28,29,30,31,32,33,34, which can offer a potential alternative to the traditional therapy with antiepileptic drugs. Specifically, both the epileptic humans and animal models, as well as the theoretical model^20^, have been conducted to investigate the feasibility of stimulation for aborting brain activities related to the absence seizures with SWD discharges^35^. Some results including the successful and unsuccessful applications of stimulus perturbations on the experimental and theoretical models^20^,^36^,^37^,^38^,^39^ have been reported, which implies that stimulation protocols for SWD may not yet be optimal^20^.

Additionally, recent experimental result^40^ has shown that there exists a basic circuit module of disinhibition in the mammalian cerebral cortex, which means that there are mutual effects among different inhibitory neurons with different time scales, meditated by the GABA_A_ and GABA_B_ inhibitory projections, respectively. Hence, there exist the important modulation functions of disinhibition for the cortical epileptic dynamics. In fact, various types of GABAergic interneuronal populations can shape the representation of cortical information^41^,^42^. A variety of pharmacological evidences in rats have suggested that GABA_B_ receptors play a critical role in the genesis of epileptic SWD^43^,^44^,^45^,^46^. Also, clinical experimental results have also shown that disinhibition function, i.e., the interaction between GABA_A_ and GABA_B_ receptors, can induce the theta-like activity in hippocampal formation slices^47^, adjust the extracellular glutamine and glutamate in rat hippocampus *in vivo*^48^, as well as regulate the transmitter release in cerebellar granule neurons^49^. Particularly, it has been demonstrated that the GABA_B_ receptors can be functionally coupled to the GABA_A_ receptors by using the selective GABA_A_ receptor agonist isoguvacine, hence leading to a disinhibition action of GABA_B_ receptor agonists. In addition, after blocking GABA_A_ receptors in ferret thalamic slices^50^, spindle oscillations can be transformed into slower ~3 Hz oscillations, which, like SWD, can be abated by GABA_B_ receptor antagonists. Hopefully, disinhibition control of SWD can be an another potential alternative to abort epileptic seizures, which need to be deeply explored.

Aside from the experimental studies, computational models are also important to explore the mechanisms underlying the occurrence and suppression of SWD. A number of developed mathematical models were used to describe the abnormal SWD^20^,^51^,^52^,^53^,^54^,^55^. Traditionally, periodic SWD discharges are considered as the homogeneous oscillations on the cerebral cortex. To represent the periodic SWD dynamics of the cerebral cortex, Taylor and Baier^52^ extended the two-variable ODE of Amari model^56^ by adding a second inhibitory population with a different timescale. It has been confirmed that the competing mechanisms among different neuronal populations can lead to robust periodic SWD discharges dynamics^57^ when they operate on the different timescales mediated by the inhibitory GABA_A_ and GABA_B_ receptors, respectively. Marten *et al.*^58^,^59^ also incorporated two separate inhibitory mechanisms with the different time scales of GABA_A_ and GABA_B_ inhibition in a cortical model and reported the generation of SWD. Wendling *et al.*^60^ also supposed two different inhibitory populations operating on the different time scales in a model of temporal lobe epilepsy to simulate the transition to epileptic spiking. However, thalamocortical interactions turn out to be crucial for the generation of SWD seizures in both the rodent models^61^,^62^ and humans^63^,^64^. Inspired by these findings, a thalamocortical model (see Fig. 1(a)) has been developed to investigate stimulus driven epileptic seizure abatement^20^,^54^. Whereas, in this thalamocortical model disinhibition functions among different inhibitory neuronal populations have not been mentioned^40^,^55^.

Based on the recent experimental result of Pi *et al.*^40^, Fan *et al.*^55^ proposed a modified Taylor-Baier model^51^ (see also Fig. 1(b) in ref. 55) with the addition of a second inhibitory neuronal population, and also considered the regulating function of disinhibition among different inhibitory neuronal populations. In particular, they mainly focused on the role of established competing effects of disinhibition in neuronal population of cortex, and computationally found that disinhibition may be a mechanism for the transition between absence and tonic-clonic seizures. Nevertheless, in this work they also ignored the thalamic function during epileptic absence seizure. Presently, we will develop a thalamocortical model (see Fig. 1(b)), which includes the cortical excitatory pyramidal (PY) cells and two different inhibitory interneuronal populations, IN_1_ and IN_2_, as well as the subcortical TC-RE circuit model composed of the thalamocortical relay cells (TC) and the reticular nucleus (RE)^54^. And then, we investigate the effects of stimulus perturbations on the occurrence of SWD discharges and its transition dynamics under the mutual functions between cortex and thalamus. We further study the control effect of disinhibition on the stimulus-induced SWD discharges.

## Results

**Figure 1:**
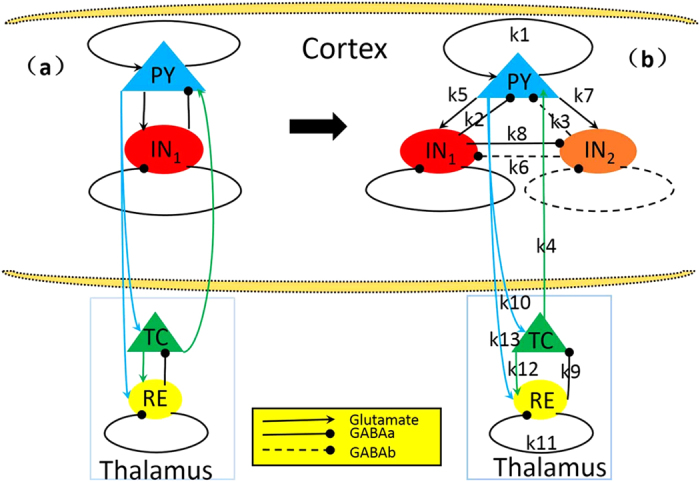
Schematic diagrams of the studied models: (**a**) the original thalamocortical model proposed by Taylor *et al.* (2014), which is composed of the cortical subnetwork consisting of the excitatory pyramidal neural population, PY, and the inhibitory interneuronal population, IN_1_, and the subcortical TC-RE circuit model mainly consists of the thalamocortical relay cells (TC) and the reticular nucleus (RE) cells (Taylor *et al.*^20^,^54^); (**b**) the modified Taylor model by introducing the second inhibitory neural population, IN_2_, into the cortical subnetwork. The proposed model involves two dependent inhibitory neural populations IN_1_ *and* IN_2_ which are mediated by the fast and slow time scales of the inhibitory receptors GABA_A_ and GABA_B_, respectively. Excitatory synaptic connections are shown in lines with arrows. Inhibitory synaptic connections are shown in lines with closed circles, where solid and dashed ones represent the fast and slow synaptic function mediated by the GABA_A_ and GABA_B_, respectively.

Based on electrophysiological experiments, we first modify the original model of Taylor^20^ (see Fig. 1(a)) by introducing the second inhibitory neuronal population, IN_2_, with a different slow timescale (see Fig. 1(b)). Thereafter, we mainly focus on the effects of interactions between the cortex and thalamus, *k*_4_ and *k*_10_, i.e., the coupling strengths from thalamus to the cortex and from cortex to thalamus, respectively, on the occurrence of the periodical spike and slow-wave discharges (SWD) and its transition dynamics. Importantly, we investigate the effect of stimulation perturbations on the generation and abortion of epileptic SWD discharges. Lastly, we consider the regulating effect of disinhibition among the two different neuronal populations, IN_1_ and IN_2_, on the occurrence or suppression of SWD discharges. In addition, it is noted that all the simulating calculations are conducted for only the PY and IN_1_, without the IN_2_ due to its much slower time scale.

### Stimulation-induced recurrent seizures of spike-wave discharges for the modified Taylor model

**Figure 2:**
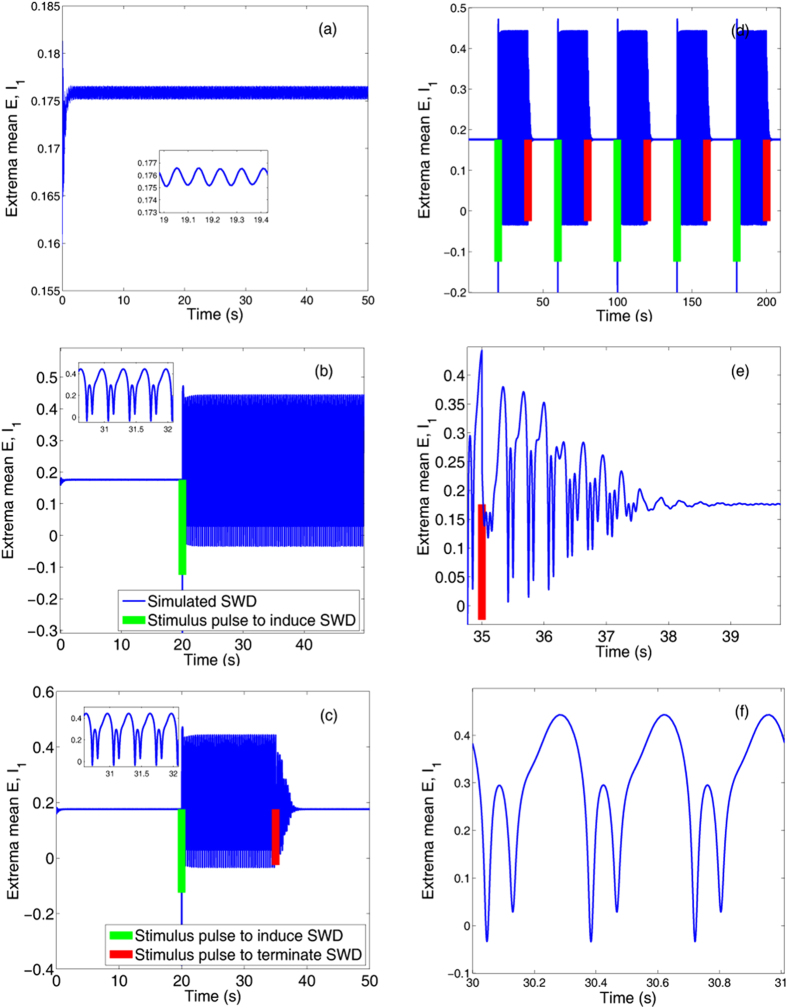
The stimulus-induced seizures of the periodical spike and wave discharge (SWD). Time series of the mean of the excitatory and inhibitory neuronal populations, PY and IN_1_: (**a**) the initial background state of the system, i.e., low-amplitude and high frequency oscillations, (**b**) SWD can be induced by the weak stimulation, −0.3, indicated by the green bar performed on the background state (**a**), (**c**) SWD can also be terminated by another successively performed weaker stimulation, −0.2, indicated by the red bar. Particularly, periodical successive stimulations can induce the recurrent seizures of SWD (**d**), (**e**,**f**) are the enlarged versions of one periodical stimulation in (**d**) describing the seizure (**f**) and seizure abatement (**e**) of SWD, respectively.

During the seizures of spike-wave discharge (SWD) the coexistence of seizure and background states has been found^20^. Also, single-pulse stimulations have been suggested to prematurely terminate the seizure (Taylor *et al.*)^20^. However, single-point stimulations or perturbations can also induce the occurrence of the SWD. As shown in the Fig. 2(a), before seizure the system shows the stable background state. From the inset of Fig. 2(a), we can see that the system can display small-amplitude and high-frequency tonic oscillations. However, as a single-point stimulation of −0.3 indicated by the green bar (Fig. 2(b)) is performed around *t* = 20 *s*, the SWD (see the inset of Fig. 2(b)) discharges can be induced from the background state. Here, the single-point stimulation can be considered as the little perturbation for the system on the background state. Interestingly, the SWD discharges can also be terminated and return to the background state when another smaller stimulation or perturbation of −0.2 (see Fig. 2(c)), indicated by the red bar, is given around *t* = 35 *s*. Hence, the SWD discharges can be induced, and also can be terminated by the single-point stimulations or perturbations, respectively.

In order to investigate the continuous effect of single-point stimulus on the dynamics of SWD discharges, we particularly perform periodic stimulations of *T* = 20 *s* from *t* = 20 *s* with the amplitudes of stimulus pulses are −0.3 and −0.2, indicated by longer green bars and shorter red bars, respectively. As seen from Fig. 2(d), periodical SWD discharges can be induced from the background states when the periodical single-point stimulus pulses (indicated by green bars) are successively performed. Also, the stimulus-induced SWD discharges can be periodically terminated and return into the background states by inputting the periodic stimulations indicated by the red bars. Therefore, under the continuous single-point stimulations the system can periodically transit between the onsets and offsets of SWD discharges, which potentially represents the epileptic recurrent seizures. Figure 2(e) is the close-up of SWD abatement in a complete stimulation period of SWD discharge, and Fig. 2(f) shows an enlargement of SWD discharges, where we can clearly see the firing frequency of 3 Hz within the SWD seizures. Because absence seizures in epilepsy is characterized by the typical 2–4 Hz SWD discharges, stimulation can biologically lead to the epileptic absence seizures and terminations.

However, during the numerical simulation of Fig. 2 we only use a single pulse stimulus with arbitrary strength and direction at the arbitrary timing of the stimulation, which is less beneficial for us to systematically understand the mechanism underlying the SWD initiation and termination by perturbation in Fig. 2(c). Following the work of Taylor *et al.*^54^, the stimulation direction in the experimental setting is not necessarily controllable, e.g., a TMS (transracial magnetic stimulation) pulse. The authors in ref. 20 (Taylor *et al.*^20^) have demonstrated that fixing the direction essentially does not restrict the generality of the investigation for the deterministic system. Therefore, during the simulations we fix the direction of the single-pulse stimulations and mainly allow their timings and amplitudes to vary.

In order to find out if the mechanism of SWD initiation and termination by perturbation in Fig. 2c is sensitive to exact timing of the stimulation, i.e. with respect to the phase of SWD, lots of numerical tests on the occurrence and termination of SWD induced by the single pulse stimulation have been done. Results have shown that typically the stimulus-initiated SWD onset may not depend on the exact timing of stimulation, i.e., with respect to the phase of SWD. By contrast, the SWD termination by perturbation of single pulse stimulation is relatively sensitive to the exact timing of the stimulations. Particularly, it is also essentially and closely correlated to the timing of the previous stimulations inducing the onset of SWD. For example, in Fig. 2(c), when changing the timing of stimulation from *t* = 35 *s* to *t* = 30 *s*, the perturbation by stimulation can not terminate the SWD anymore. However, when we change the timing of stimulation initiating the onset of SWD from *t* = 20 *s* to *t* = 10 *s*, the stimulation at *t* = 30 *s* can terminate the SWD again. Generally, in the Fig. 2(c), when the timing of stimulation inducing the SWD onset is set to *t* = 20 *s*, only on the exact timings of stimulation *t* = 28 *s*, 31 *s*, 35 *s*, 36 *s*, 37 *s*, 38 *s*, 39 *s* and 40 *s* within *t* ∈ [20 *s*, 50 *s*], the SWD can be terminated. Similarly, when changing the timing of stimulation inducing the SWD onset from *t* = 20 *s* to *t* = 10 *s*, the SWD can be terminated on the exact timings of stimulation *t* = 10 *s*, 18 *s*, 20 *s*, 21 *s*, 25 *s*, 26 *s*, 27 *s*, 28 *s*, 29 *s* and 30 *s* within *t* ∈ [10 *s*, 50 *s*]. However, it is still very difficult to figure out how sensitive the system to those particular timings of stimulation.

**Figure 3:**
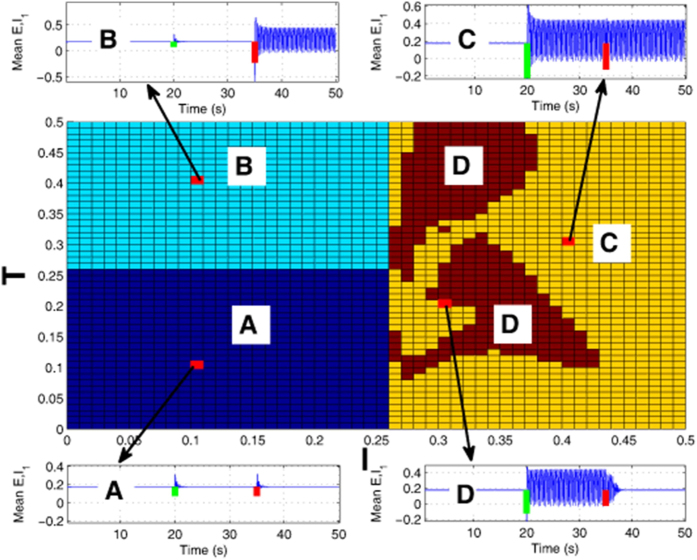
Two-dimensional plane distribution of various stimulation strengths respectively inducing (‘I’, strength of the first stimulation indicated by green bars) and terminating (‘T’, strength of the second stimulation indicated by red bars) the SWD discharges within the region [0, 0.5] × [0, 0.5]. There are four distinct regions showing the different firing states under the consecutive initiation (‘I’) and termination (‘T’) stimulations of SWD: (**A**) Both the consecutive stimulations fail to initiate the SWD. (**B**) The first stimulation fails to initiate the SWD, while the consecutive stimulation can induce the SWD. (**C**) The first stimulation can successfully induce the onset of SWD, which can not be terminated by the sequential stimulation. (**D**) The first stimulation can successfully induce the onset of SWD, which can also be terminated by the sequential stimulation.

According to the conditions of Fig. 2, in the 2-D region of Fig. 3 we want to find the typical region where the strength of first stimulation can induce the occurrence of SWDs which can also be terminated by the second stimulation. However, in order to ensure the consistency we generally use the initial letters of inducing and terminating, i.e., ‘I’ and ‘T’ to describe such an event that the SWDs can not only be induced by the first stimulation, I, but also can be terminated by sequential stimulation, T.

As to whether a stimulus with particular amplitude in the model is successful in initiating and terminating SWD, and how sensitive the system is to those particular values, without loss of generality, we investigate the robustness of system to those particular stimulation values on the basis of Fig. 2(c). Specifically, in Fig. 3 we provide the parametric distributions within the region of (I, T) ∈ [0, 0.5] × [0, 0.5], with respect to the stimulation strengths which can initiate (‘I’, green bars) and terminate (‘T’, red bars) the SWD, respectively. The horizontal and vertical axises of Fig. 3 represent the stimulation strengths of SWD initiation and termination, i.e., ‘I’ and ‘T’, represented by the initial letters of ‘initiation’ and ‘termination’ for convenience. From the Fig. 3 we can see that there are four different regions, i.e., ‘A’, ‘B’, ‘C’ and ‘D’, representing the various effects of stimulation parameter values on the SWD initiation and termination, respectively. From region ‘A’ we can see that when the stimulation strength is lower than I = T = 0.26, the SWD can not be effectively initiated by the two sequential stimulation perturbations (see the illustration corresponding to ‘A’). In the region ‘B’, where T > = 0.26, but I < 0.26, even though the SWD can not be induced by the first stimulation pulse with stimulation strength lower than I = 0.26, the immediately following stimulation pulse with stimulation strength larger than T = 0.26 can initiate the SWD. In the right side of the figure, i.e., I > 0.26, SWD can be induced by the initiation stimulation. However, in the region ‘C’, the stimulation-induced SWD can not be terminated by the consecutive stimulation perturbation. By contrast, in the region ‘D’, the stimulation-induced SWD by initiation stimulation perturbation can be effectively terminated by the sequential stimulation perturbation. Generally speaking, for the case of Fig. 2(c), only the large enough stimulation amplitude (e.g. I > = 0.26) can initiate the SWD oscillations. Once the SWD initiated, only typical abatement simulation with amplitudes of T > = 0.07 can terminate the SWD. Once the initiation stimulation is larger than I = 0.43, the SWD can not be terminated anymore.

Ultimately, the goal of abatement stimulation is to find the parameter region similar to ‘D’ in Fig. 3 to minimize the seizures. In principle, the abnormal brain activity can be restored through electrical stimulation. In epilepsy, abnormal patterns emerge intermittently^65^ which may need recurrent electrical stimulation to restore the normal brain activity. Therefore, an adaptive feedback brain stimulation control that can be applied in the necessary amount and at the specific time to suppress SWD is desirable. The finding in Fig. 2(d) that sequential single pulse stimulations induce sequential SWDs which can also be terminated by the sequential stimulations may be motivational to that effect.

### Firing transitions of modified model induced by the interactions within thalamocortical circuit

Epileptic absence and tonic-clonic seizures are primary generalized seizures involving the whole cortical region of humans^18^,^19^,^55^. Electrophysiological experiments have long revealed the existence of two-way transitions between them^18^,^19^. However, the mechanisms under the electrophysiologically observed epileptic seizures are very complex, and they can not only be caused by the cerebral cortex, but also the subcortical structures, e.g., absence seizure are known to arise in thalamo-cortical networks^61^,^62^,^63^,^64^. Experimental results have revealed that epileptic seizures and their transitions can be induced by the abnormal interactions within the corticothalamic system^61^,^62^,^63^,^64^. Based on this, computational models have been developed to study these behaviors. In this section, we further explore the transition dynamics based on the proposed thalamocortical model and mainly investigate the roles of established competing mutual functions between the cortex and thalamus for the epileptic transition dynamics.

**Figure 4:**
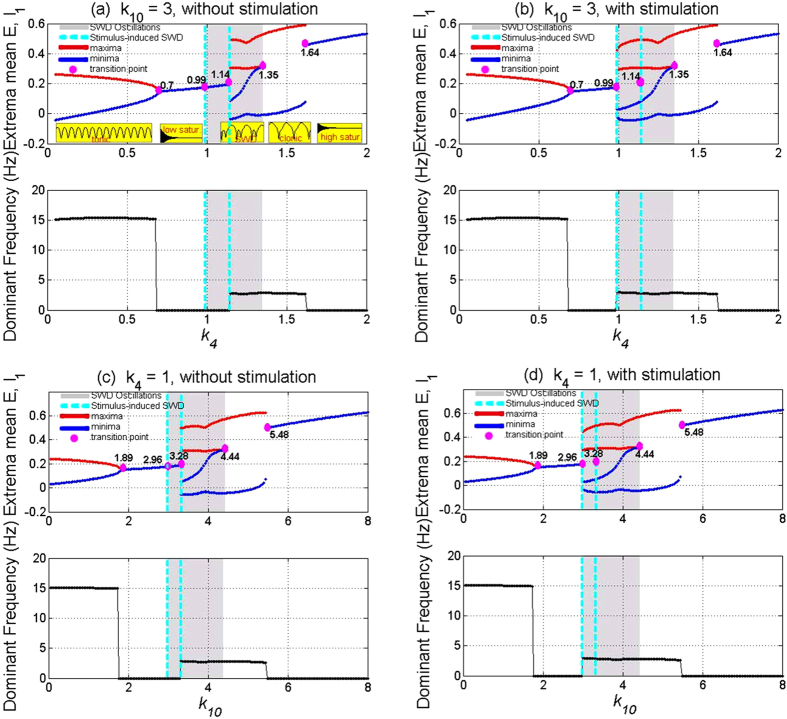
Bifurcation diagram showing the extrema of the mean of excitatory and inhibitory neuronal populations, PY and IN_1_, and their corresponding dominant frequencies. As the coupling strengths, *k*_4_, varies in the region [0, 2] with *k*_10_ = 3 (**a,b**), or *k*_10_ varies in the region [0, 8] with *k*_4_ = 1 (**c,d**), the system successively transits from the high-frequency and low-amplitude tonic oscillations (~15 Hz) to the low saturated firings, SWD discharges (~3 Hz) (shaded area), low-frequency but high-amplitude clonic oscillations (~3 Hz) and to the high saturated firing, respectively (see the illustrations in upper panels of (**a**)). Particularly, before stimulation (**a,c**) the system lies in the low saturated firings on the right short regions of *k*_4_ = 1 (**a**) and *k*_10_ = 3 (**c**). After stimulation (**b,d**), SWD can be induced from these stable low saturated firings, and the parameter regions of SWD is enlarged. The red and blue lines represent the maxima and minima, repectively. Numbered pink circles indicate the accurate bifurcation points. SWD can be typically initiated by stimulation perturbations in the regions indicated by the vertical lines.

Results of Fig. 4 show that without (Fig. 4(a,c)) and with (Fig. 4(b,d)) single-point stimulations or little perturbations projected into the system, the modified Taylor model can display rich dynamics as the coupling strength, *k*_4_ or *k*_10_, i.e., the projecting function of TC on to the PY or the feedback projecting function of PY onto the TC, varies. In particular, before stimulation, it can be seen from the upper panel of Fig. 4(a) (the bottom illustrations of the figure) that the modified Taylor model firstly transits from the simple tonic oscillation to the low saturated firing as the parameter *k*_4_ changes with fixing *k*_10_ = 3. Successively, transition to the periodic spike-wave discharges (SWD) can be found with *k*_4_ increasing to around *k*_4_ = 1.14. Afterwards, with *k*_4_ further increasing the system can transit from the SWD to the simple clonic oscillation. Finally, for the large value of *k*_4_, the system transits into the high saturated firing, which occurs around *k*_4_ = 1.64. Similarly, when we take *k*_4_ = 1, bifurcation scenario of the modified model is plotted in Fig. 4(c) as the parameter *k*_10_ varies in [0, 8]. Obviously, similar to the Fig. 4(a), rich dynamic transitions successively from simple tonic oscillation to low saturated firing, spike-wave discharge (SWD), simple clonic oscillation and to the high saturated firing can also be found in the upper panel of the Fig. 4(c). Particularly, from Fig. 4(c) we can see that as *k*_10_ increases into around *k*_10_ = 3.28, the SWD discharge can be induced. The SWD can also be terminated as *k*_10_ further increases to around *k*_10_ = 4.44, and simultaneously the systems transfer into the simple clonic oscillation.

In addition, in the lower panels of Fig. 4(a,c) we also give the variations of dominant frequencies corresponding to the various firing state transitions within the upper panels of Fig. 4(a,c). From the evolutions of dominant frequencies, we can observe one sudden dive, one sudden jump and an another sudden dive, which corresponds to the critical transitions of different firing states. Compared Fig. 4(a) with 4(c), we can see that simple tonic oscillations have the higher frequency of 15 Hz, while SWD discharge and simple clonic or slow-wave oscillations have the similar frequency of 3 Hz, which are all in line with the characteristic frequencies of electrophysiologically observed epileptic absence and tonic-clonic seizures. Hence, from Fig. 4(a,c) we can also find that with fixing *k*_4_ or *k*_10_, the small values of *k*_10_ and *k*_4_, i.e., the weak coupling strengths from PY to TC or from TC to PY, can lead to the high-frequency and low-amplitude simple tonic oscillations, moderate coupling strengths can result into the SWD discharges and low-frequency but high-amplitude simple clonic oscillations, and stronger coupling strengths can make the system into the high saturated firings.

Furthermore, in order to investigate the effect of single-point stimulation or perturbation on the dynamical transitions induced by the competing mutual functions between the cortex and thalamus, in the Fig. 4(b,d) we give the bifurcation scenari corresponding to the Fig. 4(a,c), respectively. As seen in the Fig. 4(b,d), even though we observe the similar dynamic transitions with the Fig. 4(a,c), larger parameter intervals can be found, where SWD discharges can be induced. Specifically, without the stimulation, around the right short interval of *k*_4_ = 1 indicated by the dashed lines in Fig. 4(a) the system shows the low saturated firing. However, after the introduction of stimulation (Fig. 4(b)), SWD can be induced from the low saturated firing (background state), increasing the interval of SWD discharges and accordingly decreasing the one of low saturated firing. This hold true for the case of Fig. 4(c,d), where the system shows low saturated firing within the right short interval of *k*_10_ = 3 in the Fig. 4(c) without stimulation, while SWD can also be induced by the single-point stimulation on the background low firing state, corresponding to the Fig. 4(d). It is noted that in contrast to the Fig. 4, the parameters for stimulus-induced recurrent seizures of SWD in the Fig. 2 are taken as *k*_4_ = 1 and *k*_10_ = 3, respectively.

**Figure 5:**
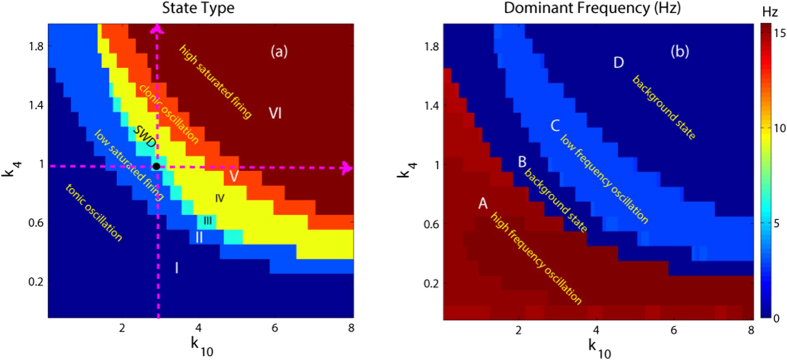
Different firing states (**a**), and variations of corresponding dominant frequency (**b**) of the modified Taylor model are shown on the parameter plane (*k*_10_, *k*_4_), where we can observe rich firing states such as simple tonic oscillation denoted by I, low saturated firings (II), stimulus-induced periodic 1-spike slow-wave discharges (SWD) (III), spontaneous SWD discharges (IV), slow-wave or simple clonic oscillations (V) and high saturated firings (VI). In addition, the state regions, I, II and VI correspond to the A, B and D regions in the frequency diagram (**b**). The region C in (**b**) can be divided into the three regions of III, IV and V in (**a**) where when we take parameters from the region III, i.e., the region of stimulus-induced SWD, the system lies in the low saturated firing before stimulation.

In order to investigate the overall transition dynamics of the modified Taylor model, as shown in Fig. 5, we depict the state transitions (see Fig. 5(a)) and corresponding variations of the dominant frequency (see Fig. 5(b)) of the proposed model as the two mutual functions between cortex and thalamus, i.e., *k*_4_ and *k*_10_, are changed. In particular, it can be seen from Fig. 5(a) that there are six different firing states, which are denoted as: I: simple high-frequency (corresponding to the region ‘A’ of Fig. 5(b)) tonic oscillations, II: low saturated firing, III: stimulus-induced periodic spike and slow-wave discharges (SWD), IV: spontaneous SWD, V: low-frequency clonic (slow-wave) oscillation and VI: high saturated firing. Accordingly, compared Fig. 5(a) with Fig. 5(b), we can find that the regions ‘A’, ‘B’ and ‘D’ in the Fig. 5(b) correspond to the regions: ‘I’, ‘II’ and ‘VI’ in Fig. 5(a), respectively, while the region ‘C’ simultaneously corresponds to the regions ‘III’, ‘IV’ and ‘V’, due to their similar firing frequencies. It should be noted that when we take parameters from the region III, i.e., the region of stimulus-induced SWD, the system displays the low saturated firing (background state) without the stimulation, being an integral part of region ‘II’ in Fig. 5(a). In addition, the vertical and horizontal pink arrows represent the two transition paths corresponding to the Fig. 4(b,d), respectively, whose crosspoint, i.e., the black point, is (*k*_4_, *k*_10_) = (1, 3) which is also used in the Fig. 2 to simulate the recurrent onsets of SWD. On the whole we can see from Fig. 5 that for the large values of *k*_4_ and *k*_10_ on the 2-D plane of (*k*_4_, *k*_10_), the system lies in the stable high saturated firing state. Physiologically, this may imply that the cortex can normally transfer the information flow into the subcortical thalamus, and simultaneously the thalamus can also reasonably relay the cortical information to feed back into the cortex. Furthermore, as *k*_4_ or *k*_10_ is taken small value, for any *k*_10_ or *k*_4_ the system basically shows the pathological epileptic seizures including the simple tonic oscillation, clonic oscillation and SWD discharges, aside from the transient background low saturated firing. This may corresponds to the conditions that the thalamus can not sufficiently relay the information from the cortex or the cortex can not normally and downstream project its information into the subcortical thalamus. Specially, on both the moderate parameters of *k*_4_ and *k*_10_, the system displays the SWD discharges including the spontaneous and stimulus-induced ones. It is worth noting that the region composed of both the stimulus-induced and spontaneous SWD demonstrates a significantly negative correlation between the mutual functions of PY and TC. This may correspond to the physiological conditions that the thalamus can redundantly receive but can not effectively relay the information from the cortex; or the thalamus can not receive the sufficient information from cortex but can redundantly and abnormally feedback the cortical information.

**Figure 6:**
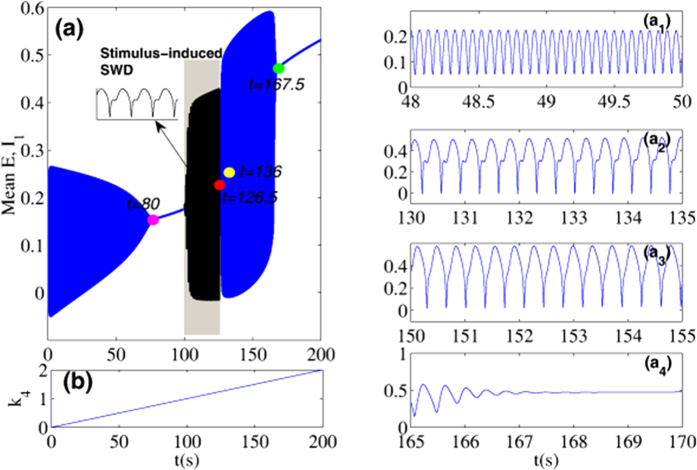
The time series for the mean of neural populations PY and IN_1_, with linearly increasing *k*_4_ from 0 to 2 and corresponding to the Fig. 4(a,b). The close-ups of the different phases of (**a**) shown in the panels (*a*_1_)-(*a*_4_): simple tonic oscillation (*a*_1_), SWD discharges (*a*_2_), simple slow-wave (clonic) oscillations (*a*_3_) and the evolution into the saturated firing from the simple slow-wave oscillations (*a*_4_). The pink, red, yellow and green solid circles represent the bifurcation critical timings of different firing states, corresponding to *t* = 80, 126.5, 136 and 167.5, respectively. The grey region corresponds to the bistable region where stimulus can induce the onsets of SWD as shown in the inset of (**a**).

It should be noted that during the bifurcation simulations (Fig. 4 and Fig. 5) same initial conditions are used for all parameters to be scanned. However, from the electrophysiological standpoint, *k*_4_ and *k*_10_, i.e., the mutual interactions between the cortex and subcortical thalamus, change continuously over time. In order to justify the bifurcation diagrams in Fig. 4 and Fig. 5 and their stability, numerical calculations should be carried out by incrementing the parameter value slightly and using the end values from the previous simulation as initial conditions for the next simulation. However, instead of being somewhat redundant, we only provide the result corresponding to the Fig. 4(a,b). And particularly for convenience and without the loss of generality, we scan k4 forwards from 0 to 2 with linear growth. As shown in Fig. 6, we first suppose that *k*_4_ is time-dependent and linearly increase over time (Fig. 6(b)). The time series can be obtained with *k*_4_ linearly increasing, shown in Fig. 6(a), where four different timings indicated by four colored circles segregate the time series into several distinct firing states. From the close-ups of the different phases of the time series, shown in the right panels of the figure (*a*_1_-*a*_4_), we can see that when *t* < ≈ 80 *s* the system displays simple tonic oscillations. In time intervals, 126.5 *s* < ≈ *t* < ≈ 136 *s* and 136 *s* < ≈ *t* < ≈ 167.5 *s*, the system displays SWD and clonic oscillations, respectively. For the rest of intervals, without the single-pulse stimulations, the system consistently shows the saturated firings. However, when the stimulation introduced, it is shown that in the grey region of Fig. 6(a), corresponding to the bistable states of the system, the SWD discharges can be induced by the single-pulse stimulation perturbations. As a whole, with *k*_4_ linearly increasing and under the single-pulse stimulus, the system shows the transitions from tonic oscillation, low saturated firing, stimulus-induced SWD, spontaneous SWD, clonic oscillations and to the high saturated firing, which is qualitatively consistent with the bifurcation diagram in Fig. 4(a,b). In addition, in order to illustrate the robustness of bifurcation dynamics in Fig. 4(a,b), we also repeat a backward scan from 2 to 0 of *k*_4_ with a linear decline. Transitions of various firings states can be typically and sequentially inversed, which somewhat reveals the bistability of system.

### The dynamical mechanisms underlying the stimulus-induced spike-wave discharges

In the last section, we have observed rich dynamics behaviors from the numerical simulations in the modified model. However, the dynamical bifurcation mechanisms underlying these transitions are still missing. Therefore, in this section, the mechanistic explanations for the bifurcation mechanisms of these transitions will be elaborated. Without loss of generality, we will still merely consider the Fig. 4(a,b) as illustrations to mechanistically understand these transitions between different dynamical behaviors.

**Figure 7:**
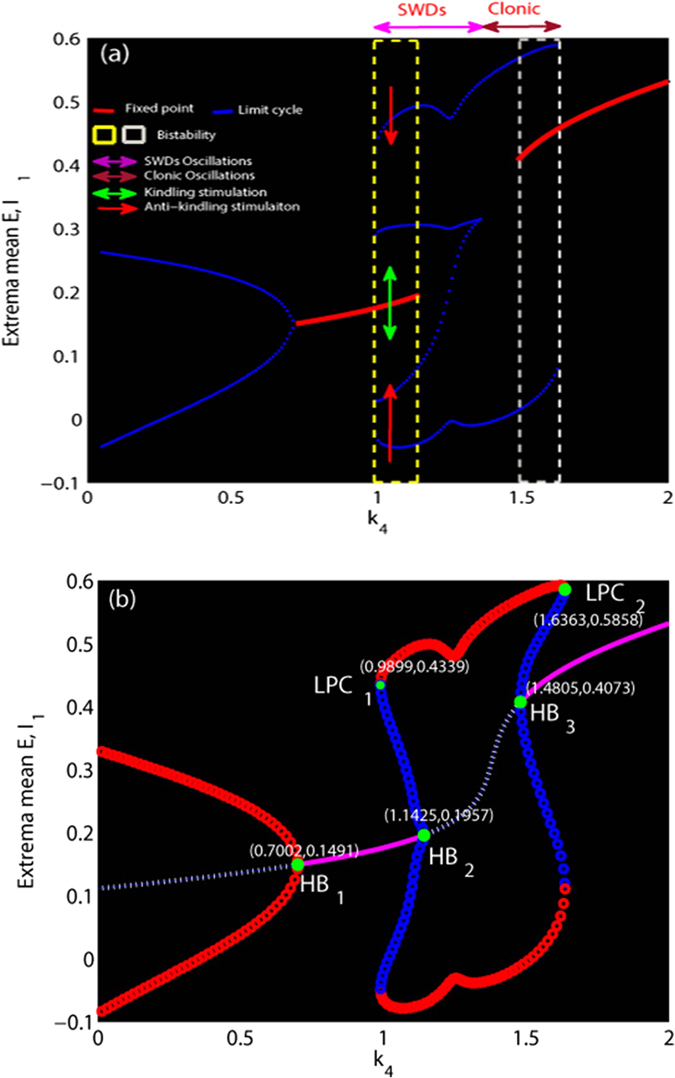
Bifurcation diagram. (**a**) Minima and maxima of time series for different values of *k*_4_. The blue and red lines represent the limit cycle and fixed point, respectively. The region indicated by yellow dashed rectangle represents the bistable parameter region between non-seizure and SWD states. The region indicated by white dashed rectangle represents the bistable region between non-seizure and clonic states. Pink and purple double arrows correspond to the SWD and clonic oscillations. Green double arrow indicates the kindling stimulation performed on the background resting state (stable focus) and the red arrow indicates the anti-kindling stimulation for the SWD discharges. (**b**) For the much small values of *k*_4_, there is one ustable focus and one stable limit cycle, all simulations converge to the simple tonic oscillations (stable limit cycle). The tonic oscillation disappears and evolves into the low saturated firings following a supercritical Hopf bifurcation (H*B*_1_). A bistable region exists with the stable focus and the stale limit cycle following a fold of cycles bifurcation (L*PC*_1_). The monostable SWD discharges and clonic oscillations exist following the subcritical Hopf bifurcation (HB_2_). Beyond the reappearance of the stable focus due to the subcritical Hopf bifurcation (HB_3_), another bistable region occurs. For much large values of *k*_4_ the bistable region disappears due to the second fold limit cycle bifurcation (LPC_2_).

In Fig. 7, the maxima and minima of the model output for different values of the parameter *k*_4_ are shown. Compared Fig. 7(a) to 7(b), we can see that for the much small values (*k*_4_ < ≈ 0.7, left side of figure) there is one unstable focus and one stable limit cycle, hence all the simulations converge to the simple tonic oscillations (stable limit cycle). For the less small values (0.7 < ≈ *k*_4_ < ≈ 0.99), the tonic oscillations of the system disappear and evolve into the low saturated firings, with only one stable focus. This follows a supercritical Hopf bifurcation at *k*_4_ ≈ 0.7 (H*B*_1_, Fig. 7(b)). However, for the less large values of *k*_4_ (0.99 < ≈ *k*_4_ < ≈ 1.14, the area of Fig. 7(a) indicated by the yellow dashed rectangle) a bistable region exists with the stable focus and the stale limit cycle. This arises following a fold of cycles bifurcation (L*PC*_1_, Fig. 7(b)) at *k*_4_ ≈ 0.99 with giving birth to one stable limit cycle and one unstable. Then the system transits from the low saturated firings to the bistable states between the non-seizure state and SWD oscillations. Consecutively, when *k*_4_ ≈ > 1.14, the stable focus loses its stability due to another subcritical Hopf bifurcation (HB_2_, Fig. 7(b)) at *k*_4_ ≈ 1.14, and the monostable SWD discharges and simple slow waves (clonic) oscillations exist in the region of 1.14 < ≈ *k*_4_ < ≈ 1.48. Beyond the reappearance of the stable focus due to the subcritical Hopf bifurcation (HB_3_, Fig. 7(b)), another bistable region indicated by the white dashed rectangle (Fig. 7(a)) between stable focus and slow wave (clonic) oscillation exists at 1.48 < ≈ *k*_4_ < ≈ 1.64. However, because of the absence of SWD, this bistable region will not be particularly elaborated. For much large values of *k*_4_ (1.64 < ≈ *k*_4_ < 2) the limit cycles disappears due to the second fold limit cycle bifurcation (LPC_2_, Fig. 7(b)) at *k*_4_ ≈ 1.64 and all the simulations converge to the steady high saturated firing state. In addition, in the region immediately preceding the bifurcation at *k*_4_ ≈ 1 complex excitable transients occur lasting several seconds.

**Figure 8:**
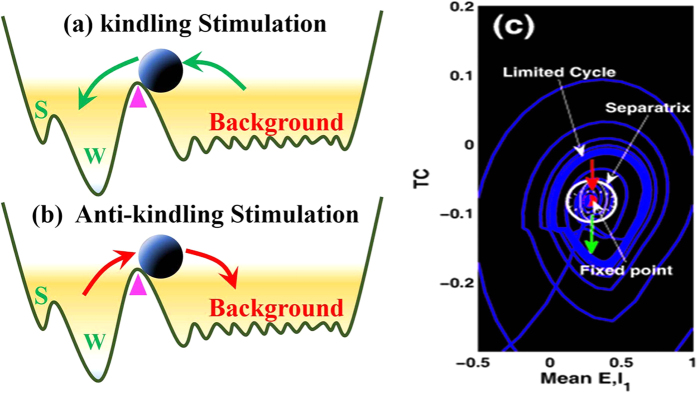
Schematic plot of the attractors modified from Hauptmann *et al*.^66^ describing the dynamical model of epileptic absence seizure characterized by Spike (‘S’) and wave (‘W’) discharges (SWD) or normal background state. (**a**) kindling (green arrows) and (**b**) anti-kindling (red arrows) stimulation can drive the neuronal population indicated by the ball from one attractor beyond the critical position (pink triangles) to another. (**c**) Phase space portrait showing the bistable region between stable focus (fixed point, red ball) and SWD (stable limit cycle). In the bistable region there exists a separating manifold indicated by the sphere (analogous to the critical positions indicated by pink triangles in (**a**,**b**)) between the two states in five dimensional state space. State transitions between the non-seizure and SWD states can occur when the stimulus indicated by the green and red arrows beyond the separatrix occurs.

In particular, the stable focus can be considered as the resting state of background EEG, and the high amplitude oscillatory attractor is analogous to the various seizure states including the epileptic SWD discharges, simple tonic and clonic (slow-wave) oscillations. A mathematically simplified double-well potential diagrams (shown in Fig. 8(a,b), modified from Hauptmann *et al.*^66^) for the complex attractors by the first approximation is considered to illustrate the impact of a basin of attraction. The first bistable region between non-seizure state (background resting state) and spike and wave discharges (SWD) can be illustrated by considering a double-well potential, where each minimum corresponds to a stable attractor surrounded by a basin of attraction. The rhythmic SWD oscillations serving as a model for epileptic absence seizures is associated with two local minima representing the spike (‘S’) and wave (‘W’) phases of SWD oscillations, while the background saturated firings with a low amplitude and high frequency oscillation serves as the non-seizure state or healthy state. The ball in Fig. 8(a,b) is used as the neuronal population. Once the system, i.e., the ball, exceeds the critical position (pink triangle shown in Fig. 8(a,b)) and enters a particular basin of attraction, it will be attracted by the corresponding attractor and ultimately relaxes into the minimum of the corresponding potential.

However, appropriate stimulation protocols can drive the ball (neuronal population) to shift from one state to another. In particular, as the ball stays in the critical region indicated by pink triangles, the future states of the system depend on the directions of stochastic disturbance. As shown in Fig. 8(a), kindling stimulation of appropriate duration indicated by the green arrows can drive the ball from the background state close to the SWD oscillation or at least into the basin of attraction of SWDs (trajectory from normal resting state to the epileptic absence seizures). By contrast, the anti-kindling stimulation of appropriate durations can also drive the neuronal population close to the background state or at least into the basin of stable focus. From the mathematical standpoint, in the bistable region of the five dimensional state space there exists a separating manifold, i.e., separatrix shown in Fig. 8(c). This can be analogous to critical positions indicated by the pink triangles in Fig. 8(a,b), even though this manifold of system is structurally complex. As soon as appropriate stimuli beyond the separatrix occur in the bistable region, transitions between the non-seizure and SWD oscillations can also occur. However, the ultimate goal of stimulation induced seizure abatement is to both maximize the duration of non-seizure state and minimize the duration of SWD oscillation.

In addition, it is should be noted that during all the simulations the initial values of five state variables are set to [0.1724, 0.1787, 0.1803, −0.0818, 0.2775]. In the first bistable parametric region between non-seizure state and SWD oscillations, the initial values for the five state variables are close to the side of separatrix near the stable focus, i.e., the system will converge to the steady states. By contrast, in the second bistable parametric region between stable focus and slow-wave clonic oscillations, the initial values are close to the side of separatrix near the stable limit cycle and the system always shows simple slow wave oscillations. What’s more, for the case of Fig. 4(c,d), the bistable region can be found in the parameter intervals of 2.96 < ≈ *k*_10_ < ≈ 3.28 indicated by the vertical dashed lines. Specially, in the 2D plane of (*k*_4_, *k*_10_), the bistable region in Fig. 5(a) corresponds to the region ‘III’.

### Inhibition-induced spike-wave discharges for the modified Taylor model

Recent experimental result (Pi *et al.*)^40^ has shown that there exists a basic circuit module of disinhibition in the mammalian cerebral cortex, which means that there are mutual effects among different inhibitory neurons with different time scales. Hence, there are the important modulation functions of disinhibition for the cortical epileptic dynamics. Actually, recent theoretical evidences based on the model of cortical neuronal populations have tentatively and numerically found that disinhibition can be a mechanism for the two-way transitions between epileptic absence and tonic-clonic seizures^55^. Motivated by these works, in the following sections we mainly consider the effects of both the inhibition and disinhibition on the onsets of SWD in the presence and absence of stimulation, respectively. Specifically, we will first investigate the inhibitory modulating effect of the introduced second inhibitory neuronal populations, IN_2_, on the initiation and abatement of SWD. Thereafter, the mechanisms of disinhibition mediated by the original first inhibitory neuronal populations, IN_1_, underlying the onsets of SWD will also be explored.

**Figure 9:**
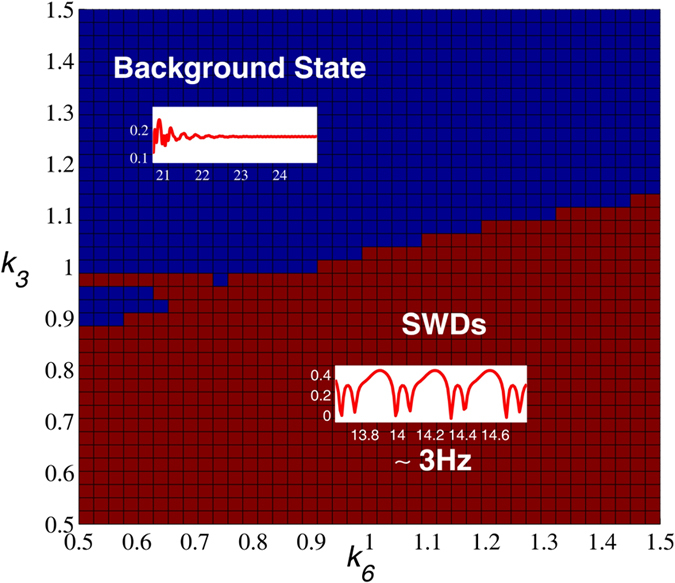
The abatement effect of the introduced neuronal population, IN_2_, on stimulation-induced SWD, mediated by the *k*_3_ and *k*_6_ parameters. The stimulus-induced ~3 Hz SWD seizure state (bottom of the figure) can be adjusted by the combined function of (*k*_3_, *k*_6_) to return into the normal background resting state (top of the figure).

In the previous sections, we have set *k*_3_ and *k*_6_, i.e., the output of the introduced second neuronal population, IN_2_, to much small values regardless of the impact from IN_2_. However, in this section, we will first investigate the abatement effect of IN_2_ on the stimulus-initiated SWD by strengthening the output of IN_2_, i.e., increasing the values of *k*_3_ and *k*_6_, which correspond to the inputs of IN_2_ into PY and IN_1_ of cortex, respectively. Without loss of generality, we take (*k*_4_, *k*_10_) = (1, 3) from the bistable region (see the region ‘III’ in Fig. 5(a)). After the initiation of SWD, we investigate the abatement effect of IN_2_ on the 2D plane of (*k*_3_, *k*_6_) ∈ [0.5, 1.5] × [0.5, 1.5], by mediating the inputs into PY and IN_1_. As shown in Fig. 9, for the small values of *k*_3_ (e.g. *k*_3_ < ≈ 1), i.e., the weak input into P*Y* from IN_2_, the stimulus-induced SWD can always not be effectively terminated by mediating the input to IN_1_ from IN_2_ for *k*_6_ ∈ [0.5, 1.5]. However, for the values of *k*_3_ larger than *k*_3_ ≈ 1, weak input into IN_1_ (e.g. *k*_6_ < ≈ 1) can start to shift the system from ~3 Hz SWD oscillation into background resting state, inducing the abatement of SWD. Particularly, as *k*_3_ getting larger, the window of SWD abatement mediated by the *k*_6_ is also gradually being enlarged. In addition, for the much large values of *k*_3_ (e.g. *k*_3_ > ≈ 1.15), the stimulus-induced SWD has been totally abated by the input into PY of IN_2_ (i.e., *k*_3_), immune to the impact of input into IN_1_ from IN_2_, with *k*_6_ ∈ [0.5, 1.5]. In sum, under the combined mediation of outputs from IN_2_ (i.e., *k*_3_ and *k*_6_) the SWD abatement effect of IN_2_ can be specifically revealed. From the mathematical standpoint, the SWD abatement effect of the IN_2_ output may be due to the shift of separatrix between the non-seizure and SWD states towards the attraction basin of stable focus, mediated by *k*_3_ and *k*_6_ in the model, with enlarging the attraction basin of stable focus and reducing the attraction basin of limit cycle symbolizing SWD. Then the initial values of the system enter the attraction basin of stable focus and the system ultimately relaxes into the background resting state.

**Figure 10:**
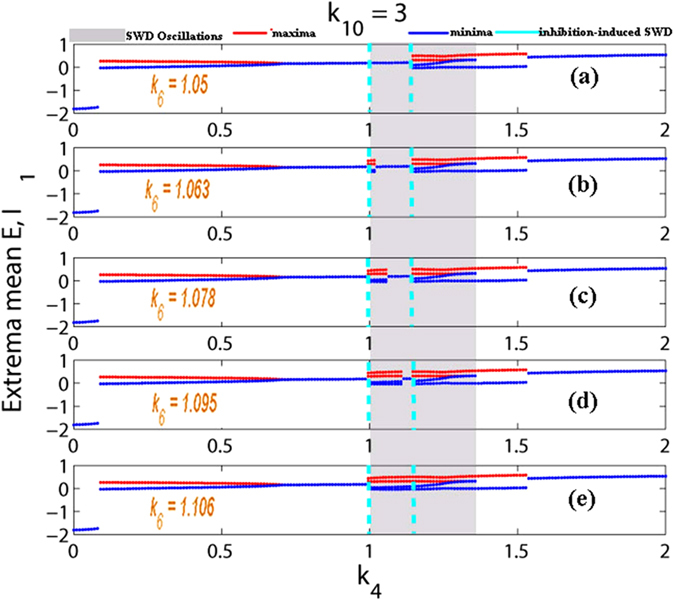
Bifurcation diagram showing the extrema of the mean of excitatory and inhibitory neuronal populations, PY and IN_1_. Inhibition-induced SWD discharges without stimulation. Corresponding to the Fig. 4(a), as the coupling strength *k*_4_ varies in the region [0, 2] with *k*_10_ = 3, with the inhibition function *k*_6_ increasing from (**a**) *k*_6_ = 1.05, (**b**) *k*_6_ = 1.063, (**c**) *k*_6_ = 1.078, (**d**) *k*_6_ = 1.095, and to (**e**) *k*_6_ = 1.106, the low saturated firings on the right short regions of *k*_4_ = 1 can be gradually disturbed into the SWD discharges. In addition, compared to the Fig. 4(a), after the introducing of inhibition function performed by the *k*_6_, as the increasing of *k*_4_, the system displays richer dynamical transition behaviors, showing the larger regions of saturated firings. Particularly, the small *k*_4_ can terminate the epileptic tonic seizures.

However, in contrast to the abatement effect of IN_2_ on the stimulus-induced SWD, we further wonder if the output from IN_2_ can also induce the onsets of SWD in the absence of stimulation, comparable to the effect of single-pulse stimulation. This may be beneficial for us to biologically understand the inner mechanisms underlying the spontaneous epileptic SWD and search the biological therapies instead of the clinical stimulation control. Therefore, in the following section we will investigate the initiation effect of IN_2_ on the SWD oscillation. Here, we take *k*_10_ = 3 with *k*_4_ varying in the interval [0, 2] and *k*_4_ = 1 with *k*_10_ varying in the interval [0, 8], respectively. As shown in the Fig. 10(a), without the impact of stimulation, i.e., as the *k*_4_ varies in [0, 2] with fixing *k*_10_ = 3, the system has a good robustness against the relatively weak inhibitory regulation function of second inhibitory neuronal population as *k*_6_ is lower around *k*_6_ = 1.05. And, on this occasion, within the right short parameter interval of *k*_4_ = 1 the system always displays the background low saturated firings (i.e., the low-amplitude and high-frequency tonic oscillations as shown in the inset of Fig. 2(a)). However, as the *k*_6_ is increased into *k*_6_ = 1.063 (Fig. 10(b)), similar to the single-point stimulation, inhibition can also induce the occurrence of SWD. With the further increasing of inhibitory function, i.e., *k*_6_ becomes larger, the larger parameter intervals can be found, where inhibition-induced SWD occurs. Compared to Fig. 10(b), as *k*_6_ is increased into *k*_6_ = 1.106 (Fig. 10(e)), enough large inhibition function can induce the occurrence of SWD on the whole parameter interval of stimulus-induced SWD. In addition, we can also see that inhibition can terminate the epileptic tonic seizures on the parameter intervals of small *k*_4_ with *k*_10_ = 3. Particularly, during the increasing of *k*_6_ saturated firing states can be repeatedly induced by the inhibition, which results in the recurrent onsets of various epileptic seizures including the simple tonic oscillations, SWD discharges and simple clonic (slow-wave) oscillations.

**Figure 11:**
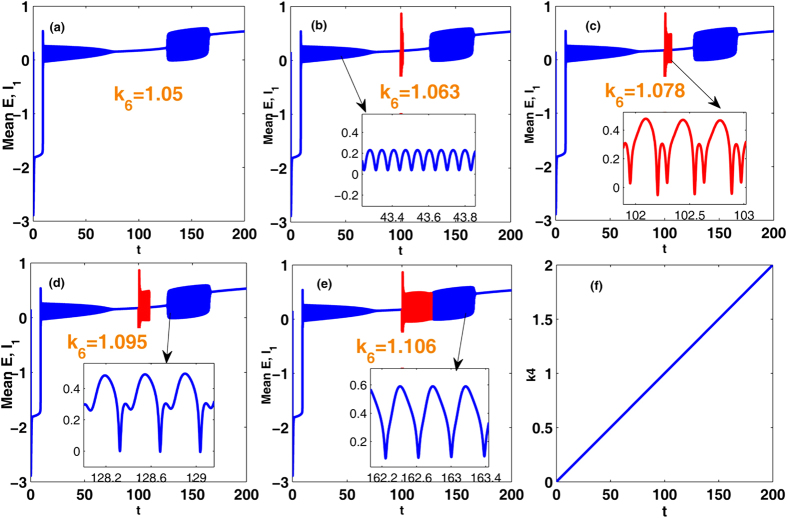
The time series for the mean of neural populations PY and IN_1_, with linearly increasing *k*_4_ from 0 to 2 (see (**f**)) and corresponding to the Fig. 10: (**a**) *k*_6_ = 1.05, (**b**) *k*_6_ = 1.063, (**c**) *k*_6_ = 1.078, (**d**) *k*_6_ = 1.095 and (**e**) *k*_6_ = 1.106. The close-ups in each subgraph respectively display the different phases of oscillating activities: simple tonic oscillation (**b**), IN_2_ induced SWD discharges (**c**), spontaneous SWD discharges (**d**) and simple slow-wave (clonic) oscillations (**e**).

Similar to Fig. 6, in the Fig. 11 we provide the time series for the mean of neural populations PY and IN_1_, with *k*_4_ linearly increasing from 0 to 2 and under the various modulating effects from the second inhibitory variable IN_2_, i.e., *k*_6_. As shown in Fig. 11(f), we suppose *k*_4_ is time-dependent and linearly increases over time. The time series respectively corresponding to the various *k*_6_ can be obtained with *k*_4_ linearly increasing. In order to more clearly observe the dynamical transitional behaviors, the insets in the subgraphs (b), (c), (d) and (e) display the different phases of oscillating activities. Particularly, instead of the stimulus, the large enough modulating functions from the second inhibitory varible IN_2_, i.e., *k*_6_, can also induce the occurrence of SWD discharges in the bistable parametric region of the system (see Fig. 7). Specially, as a whole, with the *k*_6_ gradually strengthening from (a) *k*_6_ = 1.05, (b) *k*_6_ = 1.063, (c) *k*_6_ = 1.078, (d) *k*_6_ = 1.095, and to (e) *k*_6_ = 1.106), *k*_6_-induced SWD discharges with longer time and the larger interval within the bistable region can be found.

**Figure 12:**
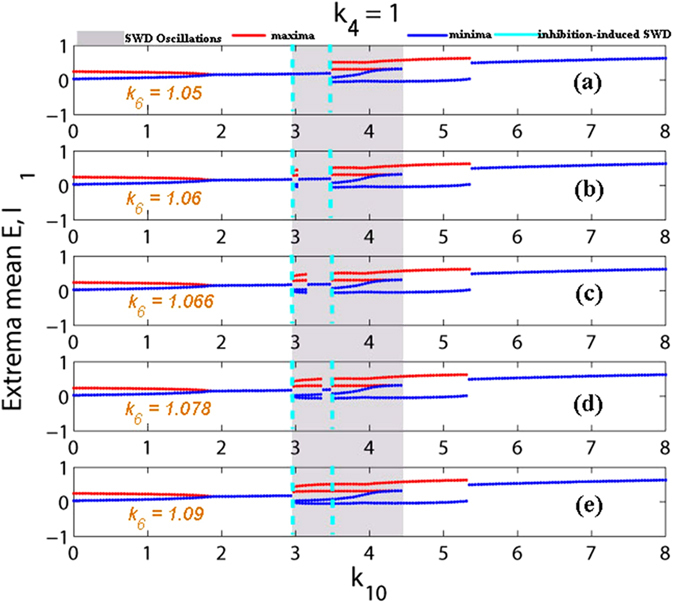
Bifurcation diagram showing the extrema of the mean of excitatory and inhibitory neuronal populations, PY and IN_1_. Inhibition-induced SWD discharges without stimulation. Corresponding to the Fig. 4(c), as the coupling strength *k*_10_ varies in the region [0, 8] with *k*_4_ = 1, with the inhibition function *k*_6_ increasing from (**a**) *k*_6_ = 1.05, (**b**) *k*_6_ = 1.06, (**c**) *k*_6_ = 1.066, (**d**) *k*_6_ = 1.078, and to (**e**) *k*_6_ = 1.09, the low saturated firings on the right short regions of *k*_10_ = 3 can be gradually disturbed into the SWD discharges. Similar to Fig. 6, compared to the Fig. 4(c), after the introducing of inhibition function performed by the *k*_6_, as the increasing of *k*_4_, the system displays richer dynamical transition behaviors, and the larger regions of saturated firings are shown.

Compared to the Fig. 10, similar scenario can be found in the Fig. 12, where we vary the *k*_10_ within [0, 8] with fixing *k*_4_ = 1, corresponding to the Fig. 4(c,d). Particularly, when *k*_6_ ≤ 1.05 (see also Fig. 12(a)), the system is basically robust to the inhibition modulation function of the second inhibitory neuronal population. However, as *k*_6_ = 1.063, corresponding to the Fig. 12(b), SWD discharges can start to be induced on the right short parameter intervals of *k*_10_ = 3 by the inhibitory function. And, the larger parameter intervals of inhibition-induced SWD can be found with the further increasing of *k*_6_ (see Fig. 12(c,d)). As shown in Fig. 12(e), as *k*_6_ is increased into *k*_6_ = 1.106, on the whole parameter interval of stimulus-induced SWD, SWD discharges can be induced by the relative larger inhibitory regulating function. Similar to the Fig. 10, saturated firing states can repeatedly occur during the increase of *k*_6_. Hence the recurrent seizures of different epileptic oscillations can be observed.

Here, it is noted that without the stimulation inhibitory modulating function of second neuronal population can also induce the occurrence of SWD, that imply the inhibition function has the similar effect with stimulation. In fact, we can see from the proposed model in Fig. 1(b) that the inhibitory function of second neuronal population, IN_2_, is actually analogous to the applied input pulse stimulation as shown in Fig. 2 indicated by the green bars. However, this inhibitory type of stimulation is of having the important feedback regulation. As shown in the cortical subnetwork, the second inhibitory neuronal population, IN_2_, can not only project inhibitory pulse inputs into both the first inhibitory neuronal population, IN_1_, and the excitatory pyramidal neurons, PY, as well as receives the feedback inhibitory action from IN_1_ and excitatory impact from PY. Compared to the stimulus-induced SWD, inhibition-induced SWD may be of having more important biological significance. In addition, we can see from Figs 10 and 12 that without the stimulation decreasing the *k*_6_ under around the *k*_6_ = 1.05 can also terminate or inhibit the occurrence of SWD discharges.

In addition, similar dynamical bifurcation schematics (see Figs 7 and 8) to the Fig. 4 can be applied to mathematically understand the mechanisms underlying the inhibition-induced SWD oscillations obtained in Figs 10 and 12. Specifically, in the absence of stimulation IN_2_ output may shift the separatrix between the non-seizure and SWD states. In particular, the increasing IN_2_ output can drive the separatrix towards the attraction basin of SWD, with enlarging the attraction basin of SWD and reducing the attraction basin of stable focus. Then the initial values of system are surrounded by the attraction basin of SWD now, and the system eventually relaxes into the SWD oscillations, comparable to the effect of stimulation.

### Disinhibition control of spike-wave dynamics for the modified Taylor model

In the last section, we investigated the inhibition regulating function of the first neuronal population, IN_1_, on the occurrence of SWD. Result shows that inhibitory modulation of IN_1_ can also induce the occurrence of SWD, which is comparable to the effect with stimulation. However, both experimental^40^ and theoretical^55^ evidences have shown the importance of disinhibition mechanisms for the generation and transition of SWD. Therefore, in this section under the inhibitory modulating effect of second inhibitory neuronal populations, IN_2_, we mainly focus on the disinhibition function of the first inhibitory neuronal populations, IN_1_, on the onsets and offsets of inhibition-induced SWD.

**Figure 13:**
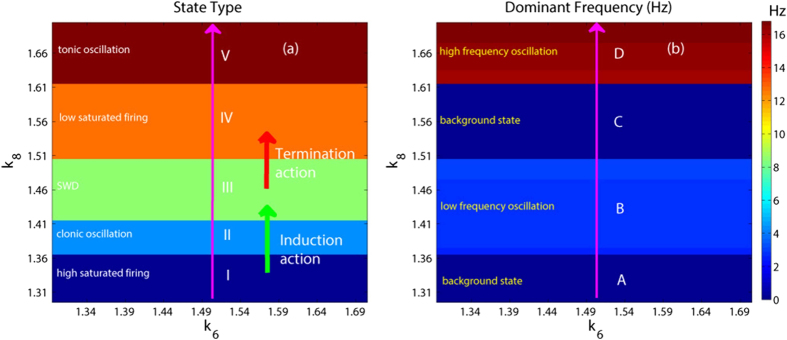
Different firing states (**a**), and variations of corresponding dominant frequency (**b**) of the modified Taylor model are shown on the parameter plane (*k*_6_, *k*_8_) within the region of [1.3, 1.7] × [1.3, 1.7], where we can observe rich firing states such as high saturated firing denoted by ‘I’, slow-wave or simple clonic oscillations (II), periodic 1-spike slow-wave discharges (SWD) (III), low saturated firing (I*V*) and simple tonic oscillation (V). It can be seen that in the local area of (*k*_6_, *k*_8_) the system is stable to the coupling strength *k*_6_, but can be relatively sensitive to the coupling strength *k*_8_. During the small region of *k*_8_ ∈  [1.3, 1.7], the system can have five times of state transitions for any *k*_6_.

**Figure 14:**
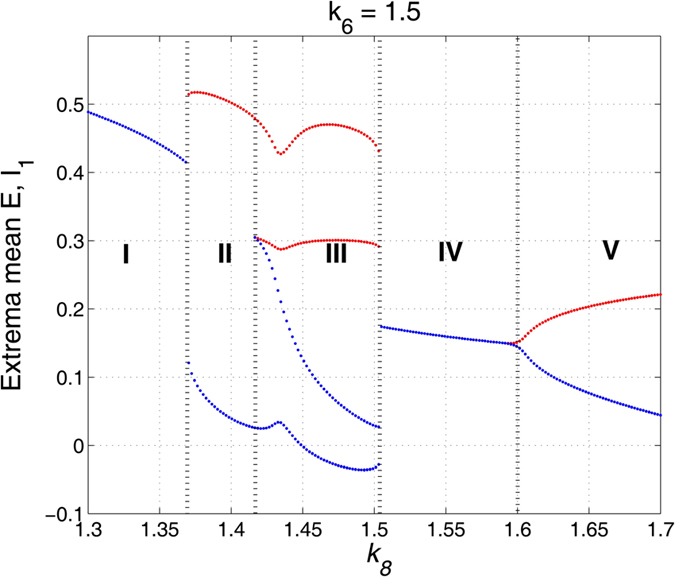
Bifurcation diagram showing the extrema of the mean of excitatory and inhibitory neuronal populations, PY and IN_1_. (**a**) The state transition diagram as the *k*_8_ varying in [1.3, 1.7] with *k*_6_ = 1.5, corresponding to the upward pink arrow in Fig. 13(a). The system transits from high saturated firing denoted by ‘I’ to clonic oscillations (II), SWD discharges (III), low saturated firing (I*V*) and to the simple tonic oscillation (V).

**Figure 15:**
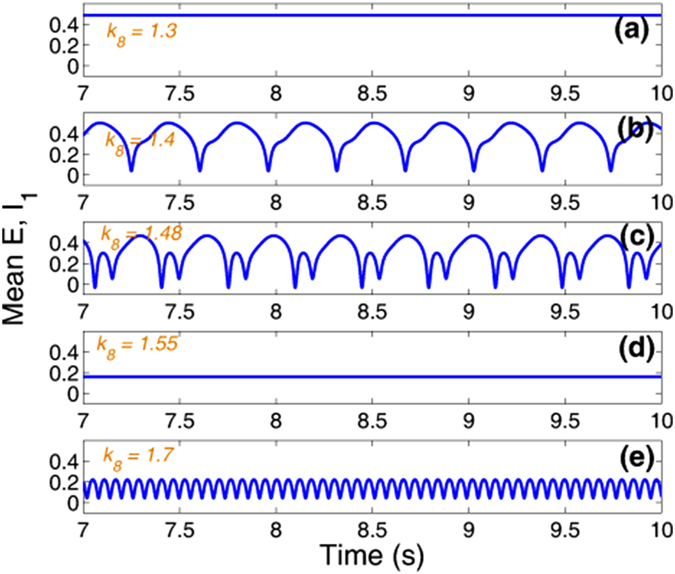
Time series of the mean of the excitatory and inhibitory neuronal populations, PY and IN_1_, corresponding to the different intervals of Fig. 14 with (**a**) *k*_8_ = 1.3 (high saturated firing), (**b**) *k*_8_ = 1.4 (clonic oscillation), (**c**) *k*_8_ = 1.48 (SWD discharges), (**d**) *k*_8_ = 1.55 (low saturated firing) and (**e**) *k*_8_ = 1.7 (tonic oscillation), respectively.

Particularly, here we take *k*_4_ = 1 and *k*_10_ = 3, as shown in Fig. 5(a) which lies in the region of stimulus-induced SWD. And importantly, we can see from Figs 10 and 12, when the inhibitory regulating function of IN_2_ is larger than *k*_6_ = 1.05, the SWD discharges can be completely induced. Hence, in order to observe the disinhibition effect of IN_1_, *k*_8_, on the inhibition-induced SWD, we here consider a local parameter region of (*k*_6_, *k*_8_), [1.3, 1.7] × [1.3, 1.7], where the critical value of *k*_6_ is larger than *k*_6_ = 1.05, which can absolutely induce the occurrence of SWD discharge. As shown in Fig. 13(a), from the bottom to top, there are five different firing states including high saturated firing indicated by (I), simple clonic oscillation (II), periodic spike-wave discharge (SWD) (III), low saturated firing (IV) and the simple tonic oscillation (V). Accordingly, the variation of corresponding dominant frequency has been given in the Fig. 13(b), where regions ‘A’, ‘C’ and ‘D’ correspond to the regions ‘I’, ‘IV’ and ‘V’, respectively. Additionally, the similar firing frequency regions ‘II’ and ‘III’ are combined into the region ‘B’. We can see from Fig. 8(a) that the system is stable to the variation of *k*_6_ within [1.3, 1.7] with any fixed *k*_8_. However, as *k*_8_ is increased from 1.3 to 1.7, for any *k*_6_ ∈ [1.3, 1.7] the system can show rich dynamic transitions from high saturated firing to clonic oscillation, SWD discharges, low saturated firing, and to tonic oscillation. For a clearer vision, corresponding to the vertical pink arrow in the Fig. 13(a), Fig. 14 depicts the 1-D dynamic transition diagram with *k*_6_ = 1.5 and *k*_8_ varying in [1.3, 1.7]. Various firing state parameter intervals corresponding to different regions of ‘I’, ‘II’, ‘III’, ‘IV’ and ‘V’ can be found. As typical examples, we also take several parameter values of (*k*_6_, *k*_8_) from the vertical pink arrow in Fig. 13(a) corresponding to the different firing states in Fig. 14, to illustrate the various firings. Figure 15 shows the typical time series of the mean for the excitatory neuronal population, PY, and inhibitory neuronal populations, IN_1_. Particularly, as *k*_6_ = 1.5, we take *k*_8_ = 1.3, 1.4, 1.48, 1.55 and 1.7, respectively. And then, the system successively displays high saturated firing corresponding to the Fig. 15(a), clonic oscillation (Fig. 15(b)), periodic spike and slow-wave discharges (SWD) (Fig. 15(c)), low saturated firing (Fig. 15(d)) and tonic oscillation corresponding to the Fig. 15(e), respectively.

More importantly, we can see from Fig. 13(a) that even though enough large inhibitory function, *k*_6_, can induce the occurrence of SWD, as *k*_8_ lies in the lower values of [1.3, 1.7], the inhibition-induced SWD can be effectively terminated into the normal high saturated firing. However, as *k*_8_ increasing into the larger values than around *k*_8_ = 1.365 and *k*_8_ = 1.415, indicated by the green upward arrow, the epileptic tonic oscillation and SWD discharges can be induced again, respectively. Because disinhibition of the first neuronal population, *k*_8_, is mediated by the inhibitory GABA_A_ receptors with the fast time scale, the disinhibition-induced SWD is in line with the experimental evidence that epileptic absence seizures characterized by 2–4 Hz periodic SWD discharge is accompanied by enhanced GABA_A_^67^. However, as the disinhibition function, *k*_8_, further increases to the larger value than *k*_8_ = 1.505 indicated by the red upward arrow, the disinhibition-induced SWD can again be terminated into the background state (low saturated state). On the other hand, due to the structurally mutual functions (see Fig. 1(b)) the increasing of *k*_8_ can, to some extent, relatively weaken the inhibition modulating effect of *k*_6_. And, as mentioned above, the inhibition function of *k*_6_ is analogous to the stimulating effect on the system, therefore this weakened inhibitory modulation of *k*_6_ is equivalent to the weaker stimulation to system. Hence, it can terminate the occurrence of SWD which corresponds to cases of Fig. 2 indicated by red bars. Above all, disinhibition can effectively control the occurrence of inhibitory regulating stimulation induced periodic spike-wave discharges.

## Discussion

Computational models provide a potential possibility for exploring the mechanisms underlying the occurrence and suppression of the typical 2–4 Hz SWD, which is the hallmark of epileptic absence seizures. Absence seizures are known to arise in thalamo-cortical networks. However, few computational evidences have devoted to the study on the thalamocortical dynamical mechanisms of epileptic SWD discharges. In addition, a variety of pharmacological^43^,^44^,^45^,^46^ and clinical^40^,^47^,^48^,^49^,^50^ evidences have shown that there exists a basic circuit module of disinhibition in the cortical neuronal network, meditated by the GABA_A_ and GABA_B_ inhibitory projections, respectively. It means that there are mutual effects among different inhibitory neurons with different time scales. Also, some results including the successful applications of stimulus-induced abatement of SWDs have been reported^38^,^39^. Therefore, to investigate the thalamocortical mechanism of SWD as well as its potential control mechanisms by stimulation and disinhibition, we developed an improved thalamocortical dynamics models of neural populations by introducing a second inhibitory neuronal population, IN_2_, with a different timescale. Hence the modified model has the competing mutual inhibitory effects in the different neuronal populations of cortex.

Based on the proposed model we have investigated the conditions on the occurrence of SWD, and its transitions to other dynamical behaviors such as epileptic tonic and clonic seizures. We first show that under the certain parameters, which biologically and structurally describes the mutual interactions between cortex and thalamus, single-point stimulation or the perturbation for the system can both induce and terminate the occurrence of SWD from the background state and return into the background state, respectively. Particularly, periodic stimulations or perturbations can produce the recurrent seizures of SWD. However, during the numerical simulation of Fig. 2 we only use a single pulse stimulus with arbitrary strength and direction at the arbitrary timing of the stimulation, which is less beneficial for us to systematically understand the mechanism underlying the SWD initiation and termination by perturbation in Fig. 2(c). In addition, following the work of Taylor *et al.*^54^, the stimulation direction in the experimental setting is not necessarily controllable, e.g., a TMS (transracial magnetic stimulation) pulse. Therefore, in the case of TMS pulse, the problem concerning stimulation is trivial and the stimulation direction can be kept constant. In order to find out if the mechanism of SWD initiation and termination by perturbation in Fig. 2c is sensitive to exact timing of the stimulation, i.e. with respect to the phase of SWD, lots of numerical tests on the occurrence and termination of SWD induced by the single pulse stimulation have been done. Results have shown that typically the stimulus-initiated SWD onset may not depend on the exact timings of stimulation, i.e., the time phases of SWD. By contrast, the SWD termination by perturbation of single pulse stimulation is relatively sensitive to the exact timings of the stimulation. Particularly, it is also essentially and closely correlated to the timing of the previous stimulations inducing the onset of SWD. However, it is still very difficult to figure out how sensitive the system to those particular timings of stimulation. As to whether a stimulus with particular amplitude in the model is successful in initiating and terminating SWD, and how sensitive the system is to those particular values, we investigate the robustness of system to those particular stimulation values on the basis of Fig. 2(c). Specifically, in Fig. 3 we provide the parametric distributions within the region of (I, T) ∈ [0, 0.5] × [0, 0.5], with respect to the stimulation strengths which can initiate (‘I’, green bars) and terminate (‘T’, red bars) the SWD, respectively. Generally, for the case of Fig. 2(c), only the large enough stimulation amplitude (e.g. I > = 0.26) can initiate the SWD oscillations. Once the SWD initiated, only typical abatement simulation with amplitudes can terminate the SWD. Once the initiation stimulation is large enough, the SWD can not be terminated anymore.

Because experimental evidences have demonstrated that the abnormal interactions within the thalamocortical circuit play a critical role for the occurrence of SWD, we then explore the transition dynamics of the modified model on the 2-D plane, (*k*_4_, *k*_10_), of mutual functions between the cortex and thalamus. The obtained results show that the proposed model can display various dynamical behaviors including the simple high-frequency and low-amplitude tonic oscillation, low saturated firing describing the background state, periodic spike and slow-wave (SWD) discharges, simple low-frequency and high-amplitude clonic oscillations. And also, it is found that 2–4 Hz SWD discharges as well as the simple tonic and clonic oscillations can be captured by the modified model which represent the typical generalized epileptic wave seizures, i.e., absence seizures, tonic seizures and clonic seizures, respectively. More importantly, the region of stimulus-induced SWD on the plane of (*k*_4_, *k*_10_) can be found, where the system lies in the background low saturated firing without single-point stimulation. Furthermore, by and large, it can be concluded that both large values of (*k*_4_, *k*_10_) can drive the system into the normal high saturated firing, which implies that the cortex can downstream transfer the cortical information into the subcortical thalamus, as well as the information from the cortex can be normally relayed by the thalamus. By contrast, the lower values of *k*_4_ or *k*_10_ can all make the cortex show the pathological rhythms, which implies the abnormal interactions between the thalamocortical circuit.

Rich dynamics behaviors can be observed from the numerical simulations in the modified model. However, the dynamical bifurcation mechanisms underlying these transitions are still missing. Without loss of generality, we considered the Fig. 4(a,b) as illustrations to mechanistically understand these transitions between different dynamical behaviors. Specifically, the system sequentially undergoes different dynamical bifurcation during the transitions. Particularly, a bistable region between the non-seizure state and SWD oscillations exists, with a stable focus and a stale limit cycle. This arises following a fold of cycles bifurcation. The stable focus can be considered as the resting state of background EEG, and the high amplitude oscillatory attractor is analogous to the various seizure states including the epileptic SWD discharges, simple tonic and clonic (slow-wave) oscillations. A mathematically simplified double-well potential diagram (shown in Fig. 8(a,b), modified from Hauptmann *et al.*^66^) for the complex attractors by the first approximation is considered to illustrate the impact of a basin of attraction, where each minimum corresponds to a stable attractor surrounded by a basin of attraction. The rhythmic SWD oscillations serving as a model for epileptic absence seizures is associated with two local minima representing the spike (S) and wave (W) phases of SWD oscillations, while the background saturated firings with a low amplitude and high frequency oscillation serves as the non-seizure state or healthy state. The ball in Fig. 8(a,b) is used as the neuronal population. Once the system, i.e., the ball, exceeds the critical position (pink triangle shown in Fig. 8(a,b)) and enters a particular basin of attraction, it will be attracted by the corresponding attractor and ultimately relaxes into the minimum of the corresponding potential. From the mathematical standpoint, in the bistable region of the five dimensional state space there exists a separating manifold, i.e., separatrix shown in Fig. 8(c). This can be analogous to critical positions indicated by the pink triangles in Fig. 8(a,b), even though this manifold of system is structurally complex. As soon as appropriate stimuli beyond the separatrix occur in the bistable region, transitions between the non-seizure and SWD oscillations can also occur. However, the ultimate goal of stimulation induced seizure abatement is to both maximize the duration of non-seizure state and minimize the duration of SWD oscillation.

In this paper we have tried our best to give the reasonable dynamical explanations for the bifurcation diagrams, as well as the possible clinical implications of the results we show. Therein, we mainly consider the mechanism of bistability^53^,^54^,^68^ to investigate the initiation and determination of SWD. However, excitability^69^ may be another critical mechanism underlying the stimulus driven seizure initiation and termination, which should be deeply investigated. In general, neurons are excitable because they are near bifurcations (transitions) from resting to spiking activity, corresponding to the qualitative change of phase portrait of the system. In this sense, the initiations and abatements of SWD may be related to change of the parameter k4, which leads to the transitions of system between stable focus and stable limit cycle. By way of comparison, the bistability mechanism underlying the stimulus-driven initiation and termination of SWD is more related to the initial conditions or the quantitative changes of the state space driven by the stimulation perturbation; but the excitability mechanism is more likely related to the changes of bifurcation parameter k4 driven by the stimulation perturbation, which can induce the qualitative transitions of different dynamic states.

In addition, we explored the effect of output from the introduced IN_2_ on the abatement effect of SWD. In sum, under the combined mediation of outputs from IN_2_ (i.e., *k*_3_ and *k*_6_) the SWD abatement effect of IN_2_ can be specifically revealed. From the mathematical standpoint, the SWD abatement effect of the IN_2_ output may be due to shift of separatrix between the non-seizure and SWD states towards the attraction basin of stable focus, mediated by *k*_3_ and *k*_6_ in the model, with enlarging the attraction basin of stable focus and reducing the attraction basin of limit cycle symbolizing SWD. Then the initial values of the system enter the attraction basin of stable focus and the system ultimately relaxes into the background resting state. What’s more, we also investigate the functions of disinhibition between the two different neuronal populations, IN_1_ and IN_2_, for the occurrence of SWD and its transitions. We first interestingly observed that without stimulation, on the parameter region of stimulus-induced SWD the inhibition regulating effect of the introduced second neuronal population, IN_2_, can also induce the occurrence of SWD, which is comparable to the effect with the stimulation. More importantly, this inhibition-induced SWD is biologically and structurally based on the feedback regulating function of IN_2_ for the PY and IN_1_. Moreover, under the inhibitory regulating function of IN_2_, increasing the disinhibition coupling function of IN_1_ can terminate the SWD discharges. Due to the relatively weakened inhibition of *k*_6_ accompanied by the increase of disinhibition of *k*_8_, the disinhibition-induced termination of SWD can also be analogous to the stimulus-induced termination of SWD. Mathematically, in the absence of stimulation IN_2_ output may shift the separatrix between the non-seizure and SWD states. In particular, the increasing IN_2_ output can drive the separatrix towards the attraction basin of SWD, with enlarging the attraction basin of SWD and reducing the attraction basin of stable focus. Then the initial values of system are surrounded by the attraction basin of SWD now, and the system eventually relaxes into the SWD oscillations, comparable to the effect of stimulation.

However, various types of GABAergic interneuronal populations can shape the representation of cortical information. Many experimental data have revealed that interneurons might be mediated by mixed GABA_A_ and GABA_B_ receptors. Also, two distinct functions of inhibition, subtractive and divisive inhibition, within the neuronal network of cortex have been suggested in the experiments^41^,^42^,^70^,^71^,^72^. In addition, it is thought that in some areas of the cortex feedforward inhibition from T*C* to I*N* is even stronger than T*C* to PY. Therefore, the addition of this feedforward inhibition is very reasonable^73^,^74^,^75^,^76^ and it should be much better computationally justified by building more realistic models. In particular, there is evidence that detailed structural connectivity within the cerebral cortex and connections among different cortex areas and thalamus nuclei may also significantly impact the system dynamics^77^,^78^,^79^,^80^,^81^. It is shown that the degree of abnormal connectivity within the thalamocortical structure is related to disease severity^81^. What’s more, in the future more realistic noise-driven stochastic dynamical systems should be explored, where noise may contribute to the termination of SWD and affect the effect of stimulation on the abatement of SWD.

Furthermore, neural system is a complex network composed of multi-layer nervous structures including the cerebral cortex and subcortical structure. e.g., basal ganglia has been demonstrated to play a key regulation function in the absence seizures^70^,^82^,^83^. According to recent studies, correlated oscillatory activity and synchronization evolutions in neuronal populations of the cerebral cortex, thalamus and basal ganglia are closely related to the generation and propagation of epileptic symptoms. A comparison of the normal and patients with epilepsy symptoms indicates that the connection loop of basal ganglia-thalamocortical is different. Hence, reasonable computational models should be further developed to uncover the detailed mechanisms of epileptic SWD discharges and improve the understanding of controlling epileptic absence seizure. What’s more, the large-scale brain modeling is a promising platform to investigate many brain disorders. Neural system is essentially a large-scale, as well as multi-scale complex system through the anatomical and electrophysiological observations. In this paper we have used the neural field model to describe the large-scale dynamics of neuronal populations within the cerebral cortex that reproduces the realistic firing rates for each neuronal population.

To summarize, in our previous work^55^ based on the cortical neural field model, we have numerically found that disinhibition can be a mechanism for the transitions between epileptic absence and tonic-clonic seizures. In this paper, we further explore the control effect of disinhibition on the occurrence and abatement of SWD. Our results about the disinhibition control of epileptic absence seizures can give some theoretical insights into understanding primary generalized epileptic seizures. However, the mechanisms under the electrophysiologically observed SWD discharges and its transitions are very complex, and should be further explored in future. More realistic computational models should be further developed to uncover the detailed mechanisms of epileptic SWD discharges and improve the understanding of controlling epileptic absence seizure.

## Materials and Methods

### Network structure

Taylor *et al.*^20^ developed a thalamocortical model (see Fig. 1(a)) to investigate effects of the stimulation on spike-wave discharge (SWD). However, in this model disinhibition functions among different inhibitory neuronal populations were not mentioned, which has been experimentally demonstrated to be crucial for the modulation of cortical network dynamics^40^. Motivated by this, we proposed a modified thalamocortical model (see Fig. 1(b)) by introducing a second inhibitory neuronal population, to explore the control effect of disinhibition on the stimulus-induced SWD discharges. Hence, the modified model is composed of the cortical excitatory pyramidal (PY) cells and two different inhibitory interneuronal populations, IN_1_ and IN_2_, with different timescales, as well as the subcortical TC-RE circuit model composed of the thalamocortical relay (TC) neurons and neurons located in the reticular nucleus (RE)^20^,^54^. The lines with arrows represent the excitatory projections mediated by glutamate. The solid and dashed lines with closed circles denote the GABA_A_–and GABA_B_–mediated inhibitory projections, respectively.

### Neural field model

Neural field model was proposed to describe the macroscopic dynamics of neural populations in an effective way with low computational cost. After the introduction of the second inhibitory interneuronal population, IN_2_, the resultant governing equations for the modified Taylor neural field model can be described as follows,


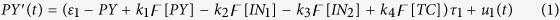



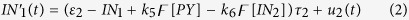



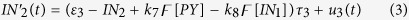



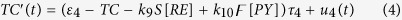



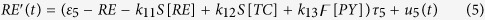


where P*Y* represents the excitatory pyramidal neuronal population, IN_1_ and IN_2_ represent the two different types of inhibitory interneuronal populations with two different fast and slow time scales determined by the parameters *τ*_2_ and *τ*_3_, respectively. *ε*_1_, *ε*_2_...*ε*_5_ are the additive constants as used in the original version of Taylor model, *k*_1_, *k*_2_...*k*_13_ are the connectivity strengths within different neuronal populations whose linking rules are in agreement with the experimentally known connections^84^ (also see Fig. 1). 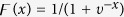 is the sigmoid transition function as used in model of Taylor^20^,^54^, where *υ* determines the steepness and *x* = PY, IN_1_, IN_2_, T*C* and R*E*.

In particular, in order to make analysis simpler and also without qualitatively impacting the related dynamics, we simplified the thalamic subsystem by replacing the sigmoid function 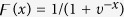 with a linear activation term *S(x*) = *αx* + *β*, where *x* = TC and RE. This also follows the connection schematic as shown in the Taylor models^20^,^54^,^84^.

### Model parameters

Most parameters in our modified Taylor model refer to the literatures^51^,^52^,^53^,^54^,^55^ (see also the Table 1) which were originally estimated from the experimental data. Due to lack of quantitative data, the mutual functional actions between the second inhibitory interneuronal population, IN_2_, and the other neuronal populations are reasonably estimated in the following numerical studies which are comparable with the original inhibitory interneuronal population, IN_2_. However, because IN_1_ and IN_2_ have the different fast and slow timescale characteristics determined by the parameters *τ*_2_ and *τ*_3_, respectively, a much smaller value of *τ*_3_ than *τ*_2_ is used to describe the dynamics equation of IN_2_.

In addition, in order to simulate the effect of stimulus-induced SWD, we performed a stimulus control u(t), i.e., a perturbation, on the cortical variables, PY and IN, in the state space, where u_1_(t) and u_2_(t) are taken some certain values (see Table 1), and (*u*_3_(t), *u*_4_(t), *u*_5_(t)) = (0, 0, 0). The control parameters are similar to those used in Taylor *et al.*^20^. During the simulations, the values of *k*_2_, *k*_3_, *k*_4_, *k*_6_, *k*_8_ and *k*_10_ vary in the physiologically reasonable range, the other parameter values in this paper follow the previous work^20^ (see also the Table 2).

### Simulation method and data analysis

During the numerical simulations for our present model, the differential equations are solved by the standard fourth-order Runge-Kutta integration scheme under the MATLAB (MathWorks, USA) simulating environment. The temporal resolution of numerical integration is fixed at 1 ms. All simulations are performed up to 20 s and the data of the stable state after a time interval of 5 s are used for analysis. Both the bifurcation and frequency analysis^55^,^85^,^86^ are utilized to characterize the critical state transitions and neural oscillations generated by our model. Firstly, to explore the transitions between various dynamical states, the bifurcation analysis is performed for several critical cortical parameters. The bifurcation diagrams are plotted by calculating the stable local minimum and maximum values of the mean for the cortical excitatory and inhibitory neuronal populations, with gradually changing these critical parameters. More importantly, to evaluate the dominant frequency of neural oscillations, the power spectral density (PSD) is estimated using the fast Fourier transform (FFT) for the time series of the mean for the cortical excitatory and inhibitory neuronal populations. Then the maximum peak frequency is defined as the dominant frequency of neural oscillations. By combining both the bifurcation and frequency analysis techniques, the typical 2–4 Hz SWD oscillation regions including the stimulus-induced and spontaneous ones can be roughly outlined in the 2-D parameter space (e.g., see Fig. 5(a)). In addition, we provide the possible dynamical mechanisms underlying the various state transitions, using the continuation package, AUTO in XPPAUT (available from http://www.math.pitt.edu/ bard/xpp/xpp.html), a tool for simulating, animating, and analyzing dynamical systems. Through the dynamical bifurcation diagram we can better understand the intrinsic transitional dynamical nature of the different oscillating activities.

## Additional Information

**How to cite this article**: Fan, D. *et al.* Stimulus-induced Epileptic Spike-Wave Discharges in Thalamocortical Model with Disinhibition. *Sci. Rep.* **6**, 37703; doi: 10.1038/srep37703 (2016).

**Publisher's note:** Springer Nature remains neutral with regard to jurisdictional claims in published maps and institutional affiliations.

## Figures and Tables

**Table 1:** Various parametric values for the neuronal populations, PY, IN_1_, IN_2_, TC and RE.

Input Constants					Time Scales				
*ε*_1_	*ε*_2_	*ε*_3_	*ε*_4_	*ε*_5_	*τ*_1_	*τ*_2_	*τ*_3_	*τ*_4_	*τ*_5_
−0.35	−3.4	−4.4	−2.0	−5	26	26 × 1.25	26 × 0.005	26 × 0.1	26 × 0.1
**Control inputs**					**Transitional Function**				
***u***_**1**_(***t***)	***u***_**2**_(***t***)	***u***_**3**_(***t***)	***u***_**4**_(***t***)	***u***_**5**_(***t***)	***υ***	***α***	***β***		
−0.3/−0.2	−0.3/−0.2	0	0	0	2.5e + 5	2.8	0.5		

PY: Excitatory pyramidal neurons; IN_1_: First inhibitory interneurons; IN_2_: Second inhibitory interneurons; TC: Thalamocortical relay cells; RE: Reticular nucleus.

**Table 2:** Thirteen various coupling strengths, *k*_1_, ..., *k*_13_, among the five different neuronal populations, PY, IN_1_, IN_2_, TC and RE, applied in the Figs 2, ..., 15, respectively.

Coupling Strength											
Symbol	Source	Target	Fig2/3	Fig4*ab*/*cd*	Fig5	Fig6/7	Fig9	Fig10/11	Fig12	Fig13/14	Fig15a–15e
*k*_1_	PY	PY	1.8	—	—	—	—	—	—	—	—
*k*_2_	IN_1_	PY	1.5	—	—	—	—	—	—	1.3–1.7	1.3–1.7
*k*_3_	IN_2_	PY	0.03	—	—	—	0.5–1.5	1.05–1.106	1.05–1.09	1.3–1.7/1.5	1.5
*k*_4_	TC	PY	1	0–2/-	0–2	0–2	—	0–2	—	—	—
*k*_5_	PY	IN_1_	4	—	—	—	—	—	—	—	—
*k*_6_	IN_2_	IN_1_	0.03	—	—	—	0.5–1.5	1.05–1.106	1.05–1.09	1.3–1.7/1.5	1.5
*k*_7_	PY	IN_2_	3	—	—	—	—	—	—	—	—
*k*_8_	IN_1_	IN_2_	1.5	—	—	—	—	—	—	1.3–1.7	1.3–1.7
*k*_9_	RE	TC	0.6	—	—	—	—	—	—	—	—
*k*_10_	PY	TC	3	−/0–8	0–8	—	—	—	0–8	—	—
*k*_11_	RE	RE	0.2	—	—	—	—	—	—	—	—
*k*_12_	TC	RE	10.5	—	—	—	—	—	—	—	—
*k*_13_	PY	RE	3	—	—	—	—	—	—	—	—

^1^PY: Excitatory pyramidal neurons; IN_1_: First inhibitory interneurons; IN_2_: Second inhibitory interneurons; TC: Thalamocortical relay cells; RE: Reticular nucleus. ^2^ “—” represents the values same to the Fig. 2.

## References

[b1] Berman R. *et al.* Simultaneous EEG, fMRI, and behavior in typical childhood absence seizures. Epilepsia 51, 2011–2022 (2010). 20608963 10.1111/j.1528-1167.2010.02652.x PMC2953613

[b2] Bai X. *et al.* Dynamic time course of typical childhood absence seizures: EEG, behavior, and functional magnetic resonance imaging. J. Neurosci. 30, 5884–5893 (2010). 20427649 10.1523/JNEUROSCI.5101-09.2010 PMC2946206

[b3] Crunelli V. & Leresche N. Childhood absence epilepsy: genes, channels, neurons and networks. Nat. Rev. Neurosci. 3, 371–382 (2002). 11988776 10.1038/nrn811

[b4] Salek-Haddadi A. *et al.* Functional magnetic resonance imaging of human absence seizures. Ann. Neurol. 53, 663–667 (2003). 12731002 10.1002/ana.10586

[b5] Sadleir L. G., Farrell K., Smith S., Connolly M. B. & Scheffer I. E. Electroclinical features of absence seizures in childhood absence epilepsy. Neurology 67, 413–418 (2006). 16894100 10.1212/01.wnl.0000228257.60184.82

[b6] Holmes G. L., McKeever M. & Adamson M. Absence seizures in children: clinical and electroencephalographic features. Ann. Neurol. 21, 268–273 (1987). 3111345 10.1002/ana.410210308

[b7] Snead O. C. Basic mechanisms of generalized absence seizures. Ann. Neurol. 37, 146–157 (1995). 7847856 10.1002/ana.410370204

[b8] Engel J., Pedley T. A. & Aicardi J. Epilepsy: a comprehensive textbook. (Lippincott Williams & Wilkins, Philadelphia, USA, 2008).

[b9] French J. *et al.* Adjunctive Perampanel for the Treatment of Drug-Resistant Primary Generalized Tonic-Clonic (PGTC) Seizures in Patients with Idiopathic Generalized Epilepsy (IGE): A Double-Blind, Randomized, Placebo-Controlled Phase III Trial (S31. 007). Neurology 84, S31–007 (2015).

[b10] Shinnar S. *et al.* Long-term outcomes of generalized tonic-clonic seizures in a childhood absence epilepsy trial. Neurology 85, 1108–1114 (2015). 26311751 10.1212/WNL.0000000000001971 PMC4603882

[b11] French J. A. *et al.* Perampanel for tonic-clonic seizures in idiopathic generalized epilepsy A randomized trial. Neurology 85, 950–957 (2015). 26296511 10.1212/WNL.0000000000001930 PMC4567458

[b12] Pilge S. Epileptiform electroencephalographic activity and generalized tonic-clonic seizures: case report. Reactions Weekly 1538, 214–214 (2015).

[b13] Gao J. *et al.* Microstructural brain abnormalities of children of idiopathic generalized epilepsy with generalized tonic-clonic seizure: A voxel-based diffusional kurtosis imaging study. J. Magn. Reson. Imaging 41, 1088–1095 (2015). 24797060 10.1002/jmri.24647

[b14] Dobesberger J. *et al.* Duration of focal complex, secondarily generalized tonic-clonic, and primarily generalized tonic-clonic seizures-A video-EEG analysis. Epilepsy Behav. 49, 111–117 (2015). 25935513 10.1016/j.yebeh.2015.03.023

[b15] Quiroga R. Q., Blanco S., Rosso O. A., Garcia H. & Rabinowicz A. Searching for hidden information with Gabor Transform in generalized tonic-clonic seizures. Electroencephal. Clin. Neurophysiol. 103, 434–439 (1997). 10.1016/s0013-4694(97)00031-x 9368487

[b16] Blumenfeld H. & Meador K. J. Consciousness as a useful concept in epilepsy classification. Epilepsia 55, 1145–1150 (2014). 24981294 10.1111/epi.12588 PMC4149314

[b17] Ji G. J. *et al.* Generalized tonic-clonic seizures: aberrant interhemispheric functional and anatomical connectivity. Radiology 271, 839–847 (2014). 24588676 10.1148/radiol.13131638

[b18] Mayville C., Fakhoury T. & Abou-Khalil B. Absence seizures with evolution into generalized tonic-clonic activity: clinical and EEG features. Epilepsia 41, 391–394 (2000). 10756402 10.1111/j.1528-1157.2000.tb00178.x

[b19] Shih T. T. & Hirsch L. J. Tonic-Absence Seizures: An Underrecognized Seizure Type. Epilepsia 44, 461–465 (2003). 12614405 10.1046/j.1528-1157.2003.39602.x

[b20] Taylor P. N. *et al.* A computational study of stimulus driven epileptic seizure abatement. PloS One 9, e114316 (2014). 25531883 10.1371/journal.pone.0114316 PMC4273970

[b21] Barnes G. N. & Paolicchi J. M. Neuropsychiatric comorbidities in childhood absence epilepsy. Nat. Clin. Pract. Neurol. 4, 650–651 (2008). 19015658 10.1038/ncpneuro0947

[b22] Caplan R. *et al.* Childhood absence epilepsy:behavioral, cognitive, and linguistic comorbidities. Epilepsia 49, 1838–1846 (2008). 18557780 10.1111/j.1528-1167.2008.01680.x

[b23] Selai C., Bannister D. & Trimble M. Antiepileptic drugs and the regulation of mood and quality of life (QOL): the evidence from epilepsy. Epilepsia 46, 50–57 (2005). 10.1111/j.1528-1167.2005.463010.x 15968809

[b24] Suzuki S., Sassa K., Abe Y. & Yamanouchi H. Generalized seizure with falling and unresponsive staring provoked by somatosensory stimulation: a video-EEG study. Epileptic Disord. 17, 336–339 (2015). 26235696 10.1684/epd.2015.0761

[b25] Kile K. B., Tian N. & Durand D. M. Low frequency stimulation decreases seizure activity in a mutation model of epilepsy. Epilepsia 51, 1745–1753 (2010). 20659150 10.1111/j.1528-1167.2010.02679.x PMC3569726

[b26] Fernandez L., Gedela S., Tamber M. & Sogawa Y. Vagus nerve stimulation in children less than 3 years with medically intractable epilepsy. Epilepsy Res. 112, 37–42 (2015). 25847337 10.1016/j.eplepsyres.2015.02.009

[b27] Bikson M. *et al.* Suppression of epileptiform activity by high frequency sinusoidal fields in rat hippocampal slices. J. Physiol. 531, 181–191 (2001). 11179402 10.1111/j.1469-7793.2001.0181j.x PMC2278457

[b28] Osorio I., Overman J., Giftakis J. & Wilkinson S. B. High frequency thalamic stimulation for inoperable mesial temporal epilepsy. Epilepsia 48, 1561–1571 (2007). 17386053 10.1111/j.1528-1167.2007.01044.x

[b29] Salem K. M., Goodger L., Bowyer K., Shafafy M. & Grevitt M. P. Does transcranial stimulation for motor evoked potentials (TcMEP) worsen seizures in epileptic patients following spinal deformity surgery? Eur. Spine J. 1–5 (2015). 10.1007/s00586-015-3993-z 25976014

[b30] Chiang C. C., Lin C. C., Ju M. S. & Durand D. M. High frequency stimulation can suppress globally seizures induced by 4-AP in the rat hippocampus: An acute *in vivo* study. Brain stimul. 6, 180–189 (2013). 22621942 10.1016/j.brs.2012.04.008 PMC3624017

[b31] Vesper J. *et al.* Chronic High-Frequency Deep Brain Stimulation of the STN/SNr for Progressive Myoclonic Epilepsy. Epilepsia 48, 1984–1989 (2007). 17561948 10.1111/j.1528-1167.2007.01166.x

[b32] Blik V. Electric stimulation of the tuberomamillary nucleus affects epileptic activity and sleep-wake cycle in a genetic absence epilepsy model. Epilepsy Res. 109, 119–125 (2015). 25524851 10.1016/j.eplepsyres.2014.10.019

[b33] Schiller Y. & Yael B. Cellular mechanisms underlying antiepileptic effects of low-and high-frequency electrical stimulation in acute epilepsy in neocortical brain slices *in vitro*. J. Neurophysiol. 97, 1887–1902 (2007). 17151229 10.1152/jn.00514.2006

[b34] Su Y., Radman T., Vaynshteyn J., Parra L. C. & Bikson M. Effects of high-frequency stimulation on epileptiform activity *in vitro*: ON/OFF control paradigm. Epilepsia 49, 1586–1593 (2008). 18397296 10.1111/j.1528-1167.2008.01592.x

[b35] Al-Otaibi F. A., Hamani C. & Lozano A. M. Neuromodulation in epilepsy. Neurosurgery 69, 957–979 (2011). 21716154 10.1227/NEU.0b013e31822b30cd

[b36] Dedeurwaerdere S. *et al.* Acute vagus nerve stimulation does not suppress spike and wave discharges in genetic absence epilepsy rats from strasbourg. Epilepsy Res. 59, 191–198 (2004). 15246120 10.1016/j.eplepsyres.2004.04.005

[b37] Saillet S. *et al.* Neural adaptation to responsive stimulation: A comparison of auditory and deep brain stimulation in a rat model of absence epilepsy. Brain Stimul. 6, 241–247 (2013). 22727526 10.1016/j.brs.2012.05.009

[b38] Berenyi A., Belluscio M., Mao D. & Buzsaki G. Closed-loop control of epilepsy by transcranial electrical stimulation. Science 337, 735–737 (2012). 22879515 10.1126/science.1223154 PMC4908579

[b39] Rajna P. & Lona C. Sensory stimulation for inhibition of epileptic seizures. Epilepsia 30, 168–174 (1989). 2494042 10.1111/j.1528-1157.1989.tb05450.x

[b40] Pi H. J. *et al.* Cortical interneurons that specialize in disinhibitory control. Nature 503, 521–524 (2013). 24097352 10.1038/nature12676 PMC4017628

[b41] Doiron B., Longtin A., Berman N. & Maler L. Subtractive and divisive inhibition: effect of voltage-dependent inhibitory conductances and noise. Neural Comput. 13, 227–248 (2010). 10.1162/089976601300014691 11177434

[b42] Ayaz A. & Chance F. S. Gain modulation of neuronal responses by subtractive and divisive mechanisms of inhibition. J. Neurophysiol. 101, 958–968 (2009). 19073814 10.1152/jn.90547.2008

[b43] Hosford D. A. *et al.* The role of GABA_B_ receptor activation in absence seizures of lethargic (lh/lh) mice. Science 257, 398–401 (1992). 1321503 10.1126/science.1321503

[b44] Puigcerver A., Van Luijtenaar E., Drinkenburg W. & Coenen A. Effects of the GABA_B_ antagonist CGP-35348 on sleep-wake states, behaviour, and spike-wave discharges in old rats. Brain Res. Bull. 40, 157–162 (1996). 8736575 10.1016/0361-9230(96)00046-9

[b45] Smith K. A. & Fisher R. S. The selective GABA_B_ antagonist CGP-35348 blocks spike-wave bursts in the cholesterol synthesis rat absence epilepsy model. Brain Res. 729, 147–150 (1996). 8876982

[b46] Snead O. C. Evidence for GABA_B_-mediated mechanisms in experimental generalized absence seizures. Eur. J. Pharmacol. 213, 343–349 (1992). 1319918 10.1016/0014-2999(92)90623-c

[b47] Konopacki J., Golebiewski H., Eckersdorf B., Blaszczyk M. & Grabowski R. Theta-like activity in hippocampal formation slices: The eVect of strong disinhibition of GABA_A_ and GABA_B_ receptors. Brain Res. 775, 91–98 (1997). 9439832 10.1016/s0006-8993(97)00919-0

[b48] Kanamori K. Disinhibition reduces extracellular glutamine and elevates extracellular glutamate in rat hippocampus *in vivo*. Epilepsy Res. 114, 32–46 (2015). 26088883 10.1016/j.eplepsyres.2015.03.009 PMC4475281

[b49] Kardos J., Elster L., Damgaard I., Krogsgaard-Larsen P. & Schousboe A. Role of GABA_B_ Receptors in Intracellular Ca2 + Homeostasis and Possible Interaction Between GABA, and GABA_B_ Receptors in Regulation of Transmitter Release in Cerebellar Granule Neurons. J. Neurosci. Res. 39, 646–655 (1994). 7897700 10.1002/jnr.490390604

[b50] von Krosigk M., Bal T. & McCormick D. A. Cellular mechanisms of asynchronized oscillation in the thalamus. Science 261, 361–364 (1993). 8392750 10.1126/science.8392750

[b51] Taylor P. N. & Baier G. A spatially extended model for macroscopic spike-wave discharges. J. Comput. Neurosci. 31, 679–684 (2011). 21556886 10.1007/s10827-011-0332-1

[b52] Wang Y., Goodfellow M., Taylor P. N. & Baier G. Phase space approach for modeling of epileptic dynamics. Phys. Rev. E. 85, 061918 (2012). 10.1103/PhysRevE.85.061918 23005138

[b53] Taylor P. N. *et al.* A model of stimulus induced epileptic spike-wave discharges. 2013 IEEE Sym Comput Intel Cogn Algor Mind, and Brain (CCMB)(Singapore). (April 2013), 53–59 (2013).

[b54] Taylor P. N. *et al.* Optimal control based seizure abatement using patient derived connectivity. Front. Neurosci. 9, 00202 (2015). 10.3389/fnins.2015.00202 PMC4453481 26089775

[b55] Fan D., Wang Q. & Perc M. Disinhibition-induced transitions between absence and tonicclonic epileptic seizures. Sci. Rep. 5, 12618 (2015). 26224066 10.1038/srep12618 PMC4519733

[b56] Amari S. Dynamics of pattern formation in lateral inhibition type neural fields. Biol. Cybern. 27, 77–87 (1997). 10.1007/BF00337259 911931

[b57] Fong G. C. *et al.* Childhood absence epilepsy with tonic-clonic seizures and electroencephalogram 3-4-Hz spike and multispikes low wave complexes: linkage to chromosome 8q24. Am. J. Hum. Genet. 63, 1117–1129 (1998). 9758624 10.1086/302066 PMC1377498

[b58] Marten F., Rodrigues S., Benjamin O., Richardson M. P. & Terry J. R. Onset of polyspike complexes in a mean-field model of human electroencephalography and its application to absence epilepsy. Philos. Trans. A Math. Phys. Eng. Sci. 367, 1145–1161 (2009). 19218156 10.1098/rsta.2008.0255

[b59] Marten F., Rodrigues S., Suffczynski P., Richardson P. M. & Terry J. R. Derivation and analysis of an ordinary differential equation mean-field model for studying clinically recorded epilepsy dynamics. Phys. Rev. E Stat. Nonlin. Soft Matter Phys. 79, 021911 (2009). 19391782 10.1103/PhysRevE.79.021911

[b60] Wendling F., Bartolomei F., Bellanger J. J. & Chauvel P. Epileptic fast activity can be explained by a model of impaired GABAergic dendritic inhibition. Eur. J. Neurosci. 15, 1499–1508 (2002). 12028360 10.1046/j.1460-9568.2002.01985.x

[b61] Liu Z., Vergnes M., Depaulis A. & Marescaux C. Evidence for a critical role of GABAergic transmission within the thalamus in the genesis and control of absence seizures in the rat. Brain Res. 545, 1–7 (1991). 1650272 10.1016/0006-8993(91)91262-y

[b62] Tenney J. R., Duong T. Q., King J. A., Ludwig R. & Ferris C. F. Corticothalamic modulation during absence seizures in rats: a functional MRI assessment. Epilepsia 44, 1133–1140 (2003). 12919383 10.1046/j.1528-1157.2003.61002.x PMC2962948

[b63] Errington A. C., Cope D. W. & Crunelli V. Augmentation of Tonic GABA_A_ Inhibition in Absence Epilepsy: Therapeutic Value of Inverse Agonists at Extrasynaptic GABA_A_ Receptors. Adv. Pharm. Sci. Sep 5 (2011). 10.1155/2011/790590 PMC3168769 21912539

[b64] Moeller F. *et al.* Representation and propagation of epileptic activity in absences and generalized photoparoxysmal responses. Hum. Brain Mapp. 34, 1896–1909 (2013). 22431268 10.1002/hbm.22026 PMC6870437

[b65] Goodfellow M., Schindler K. & Baier G. Intermittent spike-wave dynamics in a heterogeneous, spatially extended neural mass model. Neuroimage 55, 920–932 (2011). 21195779 10.1016/j.neuroimage.2010.12.074

[b66] Hauptmann C. & Tass P. A. Cumulative and after-effects of short and weak coordinated reset stimulation: a modeling Study. J. Neural. Eng. 6, 016004 (2009). 19141875 10.1088/1741-2560/6/1/016004

[b67] Cope D. W. *et al.* Enhanced tonic GABA_A_ inhibition in typical absence epilepsy. Nat. Med. 15, 1392–1398 (2009). 19966779 10.1038/nm.2058 PMC2824149

[b68] Suffczynski P., Kalitzin S. & Da Silva F. H. L. Dynamics of non-convulsive epileptic phenomena modeled by a bistable neuronal network. Neurosci. 126, 467–484 (2004). 10.1016/j.neuroscience.2004.03.014 15207365

[b69] Izhikevich E. M. Dynamical systems in neuroscience: the geometry of excitability and bursting. Nature 387, 767–768 (2015).

[b70] Vercueil L. *et al.* High-frequency stimulation of the sub-thalamic nucleus suppresses absence seizures in the rat: comparison with neurotoxic lesions. Epilepsy Res. 31, 39–46 (1998). 9696299 10.1016/s0920-1211(98)00011-4

[b71] Papasavvas C. A., Wang Y., Trevelyan A. J. & Kaiser M. Gain control through divisive inhibition prevents abrupt transition to chaos in a neural mass model. Phys. Rev. E Stat. Nonlin. Soft Matter Phys. 92, 032723 (2015). 26465514 10.1103/PhysRevE.92.032723 PMC4789501

[b72] Sturgill J. F. & Isaacson J. S. Somatostatin cells regulate sensory response fidelity via subtractive inhibition in olfactory cortex. Nat. Neurosci. 18, 531–535 (2015). 25751531 10.1038/nn.3971 PMC4452122

[b73] Porter J. T., Johnson C. K. & Agmon A. Diverse types of interneurons generate thalamus-evoked feedforward inhibition in the mouse barrel cortex. J. Neurosci. 21, 2600–2710 (2001). 11306623 10.1523/JNEUROSCI.21-08-02699.2001 PMC6762510

[b74] Swadlow H. A. Thalamocortical control of feed-forward inhibition in awake somatosensory ‘barrel’ cortex. Philos. Trans. R Soc. Lond. B Biol. Sci. 357, 1717–1727 (2002). 12626006 10.1098/rstb.2002.1156 PMC1693091

[b75] Inoue T. & Imoto K. Feedforward inhibitory connections from multiple thalamic cells to multiple regular-spiking cells in layer 4 of the somatosensory cortex. J. Neurophysiol. 96, 1746–1754 (2006). 16855112 10.1152/jn.00301.2006

[b76] Delevich K., Tucciarone J., Huang Z. J. & Li B. The mediodorsal thalamus drives feedforward inhibition in the anterior cingulate cortex via parvalbumin interneurons. J. Neurosci. 35, 5743–5753 (2015). 25855185 10.1523/JNEUROSCI.4565-14.2015 PMC4388929

[b77] Volman V., Perc M. & Bazhenov M. Gap junctions and epileptic seizures-two sides of the same coin? PLoS One 6, e20572 (2011). 21655239 10.1371/journal.pone.0020572 PMC3105095

[b78] Guo D. *et al.* Firing regulation of fast-spiking interneurons by autaptic inhibition. Epl 114, 30001 (2016).

[b79] Guo D. *et al.* Regulation of irregular neuronal firing by autaptic transmission. Sci. Rep. 6, 26096 (2016). 27185280 10.1038/srep26096 PMC4869121

[b80] Sinha N., Taylor P. N., Dauwels J. & Ruths J. Development of optimal stimuli in a heterogeneous model of epileptic spike-wave oscillations. IEEE International Conference on Systems, Man, and Cybernetics. 3160–3165 (2014).

[b81] O’Muircheartaigh J. *et al.* Abnormal thalamocortical structural and functional connectivity in juvenile myoclonic epilepsy. Brain A Journal of Neurology 135, 3635–3644 (2012). 23250883 10.1093/brain/aws296 PMC3525058

[b82] Deransart C., Vercueil L., Marescaux C. & Depaulis A. The role of basal ganglia in the control of generalized absence seizures. Epilepsy Res. 32, 213–323 (1998). 9761322 10.1016/s0920-1211(98)00053-9

[b83] Rektor I., Kuba R., Brazdil M. & Chrastina J. Do the basal ganglia inhibit seizure activity in temporal lobe epilepsy? Epilepsy Behav. 25, 56–59 (2012). 22835431 10.1016/j.yebeh.2012.04.125

[b84] Pinault D. & O’Brien T. J. Cellular and network mechanisms of genetically-determined absence seizures. Thal. Relat. Syst. 3, 181–203 (2005). 10.1017/S1472928807000209 PMC3168114 21909233

[b85] Chen M. *et al.* Bidirectional control of absence seizures by the basal ganglia: a computational evidence. PLoS Comput. Biol. 10, e1003495 (2014). 24626189 10.1371/journal.pcbi.1003495 PMC3952815

[b86] Chen M. *et al.* Critical Roles of the Direct GABAergic Pallido-cortical Pathway in Controlling Absence Seizures. PLoS Comput. Biol. 11, e1004539 (2015). 26496656 10.1371/journal.pcbi.1004539 PMC4619822

